# ADAM12 in a stromal-checkpoint framework: integrating tumor microenvironment remodeling and therapy resistance in solid tumors

**DOI:** 10.1186/s12964-026-03103-8

**Published:** 2026-07-25

**Authors:** Shaochun Liu, Jian Xu, Han Zhang, Yuhan Tang, Xiaoxi Han, Wenjing Xiong, Linlin Fan, Chang Su, Zhiqi Sui, Wenhui Zhao

**Affiliations:** 1 Department of Medical Oncology, Harbin Medical University Cancer Hospital, Harbin Medical University, 150 Haping Road, Harbin, Heilongjiang 150081 China; 2 Department of Surgical Oncology, The First Affiliated Hospital of Bengbu Medical University, Bengbu, Anhui China

**Keywords:** ADAM12, Stromal-checkpoint framework, Tumor microenvironment, Extracellular matrix remodeling, Immune exclusion

## Abstract

The tumor microenvironment (TME) is now understood as an active regulator of therapeutic vulnerability. In this Review, we use an integrative stromal-checkpoint framework, informed by published stromal and fibroblast checkpoint studies, to describe stromal programs that couple extracellular matrix (ECM) remodeling, metabolic stress, vascular dysfunction, and immune exclusion into treatment-resistant tumor ecologies. We focus on a disintegrin and metalloproteinase 12 (ADAM12) because ADAM12-high or ADAM12-positive tumor-stromal states have been linked to collagen-rich desmoplasia, mechanotransduction, hypoxia-associated stress, lactate-associated acidosis, immune-excluded spatial organization, and therapy resistance in solid tumors. Evidence spans fibroblast, mesenchymal stromal-cell, and tumor-cell perturbation studies; mechanistic substrate and signaling analyses; spatial and clinical datasets; and broader tissue-level immune contexts relevant to lymphatic drainage, tumor-draining lymph node (TDLN) priming, tissue-resident immune cells, and ILC2/type 2 immune programs. Across these evidence layers, ADAM12 emerges as an isoform-aware and cell-of-origin-dependent marker, amplifier, and context-dependent candidate target for selected high-barrier tumor states. We discuss how ADAM12-informed biomarkers, spatial barrier profiling, and stromal-priming strategies can support patient stratification and combination-therapy design. Overall, ADAM12-associated biology provides a useful model for examining how stromal resistance may be built, maintained, and therapeutically remodeled in selected tumor contexts.

## Introduction

Tumors develop within complex and evolving ecosystems in which malignant cells, stromal populations, vascular elements, immune cells, and extracellular matrix (ECM) components interact continuously under conditions of hypoxia, nutrient limitation, inflammation, and mechanical stress [1]. These pressures do not merely accompany cancer progression; they actively shape treatment response by generating microenvironmental states that support tumor persistence, restrict drug delivery, and impair effective antitumor immunity [2]. Across solid tumors, one recurring principle is that stromal remodeling is not a passive consequence of disease, but a dynamic determinant of therapeutic vulnerability, particularly in tumors where microenvironmental reprogramming influences immunotherapy resistance [3].

Among stromal populations, cancer-associated fibroblasts (CAFs) have emerged as particularly influential organizers of this barrier phenotype. CAFs remodel ECM architecture, amplify transforming growth factor-β-driven fibrotic programs, alter tissue stiffness and vascular function, and secrete factors that reinforce tumor survival, immune suppression, and adaptive resistance to therapy [4–6]. CAFs are heterogeneous and plastic, differing in spatial localization, inflammatory activity, matrix production, antigen presentation, and metabolic crosstalk, as shown by single-cell studies across multiple tumor types [7–9]. This diversity places CAFs among the central regulators of tumor growth, fibrosis, immunity, and therapeutic response [10]. Seminal single-cell studies link specific fibroblast clusters and LRRC15-positive myofibroblastic states to immunotherapy resistance, positioning ADAM12-positive CAF/myCAF states within a broader CAF landscape [11, 12]. Recent spatial single-cell analysis in skin cancers further illustrates that matrix CAFs and immunomodulatory CAFs can shape tumor-immune organization through distinct mechanisms, highlighting the importance of CAF subtype, location, and immune function [13]. Breast cancer studies likewise show how CAF plasticity can generate barriers and therapeutic opportunities in a tumor-type-specific manner [14]. CAF-derived extracellular vesicles provide an additional route through which fibroblast states communicate with tumor and immune compartments [15]. This heterogeneity helps explain why stromal targeting has often produced inconsistent results: not all fibroblast states are equivalent, and not all stromal programs are equally important for sustaining treatment resistance.

This complexity motivates an integrative stromal-checkpoint framework, but the terminology requires careful qualification. Published studies have used “stromal checkpoint” to describe specific ECM- or stroma-linked immunosuppressive control nodes, including the fibronectin–ILT3 interaction that suppresses tumor-associated myeloid cells and the DDR1/collagen barrier that limits T-cell infiltration [16, 17]. In parallel, recent fibroblast-centered Perturb-seq and functional validation work identified ADAM12 as a “fibroblast checkpoint” that links TGF-β-dependent myCAF programming to restraint of IFN-I-responsive antitumor fibroblast states [18]. Building on these published uses, we use the term stromal-checkpoint framework in this Review to describe stromal programs or control nodes that stabilize tumor-supportive microenvironments by coupling three interrelated functions: physical remodeling of the ECM-rich barrier, metabolic adaptation to stress, and immune exclusion. TGF-β signal transduction exemplifies this stromal control because it connects fibrosis, cancer progression, immune suppression, and therapeutic resistance across multiple tissue settings [19]. Seminal immunotherapy studies further show why stromal TGF-β biology matters: TGF-β contributes to T-cell exclusion and attenuates response to PD-L1 blockade, whereas TGF-β-rich metastatic colon cancer models evade immunity by excluding T cells and blocking TH1-effector acquisition [20, 21]. In this view, the stromal compartment is a coordinated system that can buffer tumors against otherwise rational therapies. Such a framework is consistent with evidence that response to immunotherapy is shaped by conserved microenvironmental states and spatially organized multicellular immune hubs [22, 23]. Lymphatic remodeling can stratify melanoma immune parameters and shape immunotherapy-relevant tumor immunity [24]. Metabolic reprogramming can restore effector function in terminally exhausted CD8 + T cells in solid-tumor immunotherapy models [25]. Pancreatic cancer and other stromal-rich tumors provide particularly compelling settings for applying this model to immunotherapy resistance [3].

Within this qualified framework, ADAM12 is an informative focal point linking CAF/myCAF biology, stromal remodeling, and immune-state regulation. We therefore discuss ADAM12 as a fibroblast-checkpoint-supported stromal-barrier marker and candidate functional node, rather than as a universally established stromal checkpoint across all tumor contexts.

Accordingly, we interpret ADAM12 through an evidence-stratified lens that separates direct ADAM12 perturbation studies from mechanistic substrate or signaling support, correlative clinical datasets, and broader tissue-level contextual biology. This distinction allows hypoxia, lactate-associated acidosis, lymphatic/TDLN biology, and type 2 immune context to be discussed as relevant ADAM12-associated contexts without implying uniform ADAM12 dependence.

The significance of ADAM12 also extends beyond tissue-level biology. ADAM12 has shown potential translational value as a biomarker linked to aggressive disease behavior, stromal activation, and adverse clinical outcome across selected tumor contexts [26–28]. These clinical associations support an ADAM12-centered synthesis of stromal barrier biology and provide a rationale for discussing ADAM12-informed biomarker and stromal-priming strategies.

This Review synthesizes structural, mechanistic, spatial, and translational evidence to define how ADAM12 relates to CAF/myCAF-state regulation, ECM remodeling, fibrosis and desmoplasia, hypoxia-associated metabolic adaptation, vascular and lymphatic tissue organization, and immune-excluded tumor states.

## ADAM12 background: structural organization, isoforms, and biological functions

### Structural organization and isoform-dependent interpretation

ADAM12 is a multidomain member of the ADAM family of metalloproteinases, a group of membrane-associated or secreted protease-related proteins that combine adhesion-related domains with metalloproteinase-dependent and metalloproteinase-independent functions. In cancer and inflammation, ADAM family members act as regulated sheddases and cell-surface modulators that alter growth-factor availability, cytokine and receptor signaling, adhesion, Notch-related communication, and immune recognition [29]. The membrane-associated ADAM12-L protein contains an extracellular prodomain, metalloproteinase domain, disintegrin-like domain, cysteine-rich/EGF-like region, transmembrane segment, and cytoplasmic tail. ADAM12-S lacks the transmembrane and cytoplasmic regions and is generally secreted [30–32]. These structural differences shape function: ADAM12-L is positioned for cell-surface adhesion, integrin-associated signaling, internalization, and local sheddase events, whereas ADAM12-S contributes to soluble or extracellular protease-dependent effects in specific systems [33]. More broadly, studies of ADAM10 show how ADAM-family substrate selection depends on membrane-proximal cleavage geometry, tetraspanin-associated regulation, and ectodomain shedding of cell-surface receptors or adhesion proteins [34, 35]. ADAM17 provides another well-known comparison because it is a major inflammatory and cancer-associated sheddase whose dysregulation affects cytokine, receptor, and growth-factor signaling in multiple disease contexts [36]. In the Notch pathway, ADAM-family proteases act together with furin and γ-secretase to regulate receptor processing and downstream signaling, providing a family-level context for ADAM12-linked Notch–HB-EGF/EGFR signaling [37]. Functional analysis of a breast cancer-associated mutation in the ADAM12 intracellular domain further shows that cytoplasmic-tail context can influence ADAM12-L behavior [38]. Proteomic studies indicate that ADAM12 expression and secretion can be influenced by protein-processing and trafficking regulators, including endoplasmin and myoferlin, reinforcing the importance of maturation and secretory context [39, 40].

Isoform-aware analysis is essential because ADAM12 measurements may capture mRNA, total protein, cell-surface protein, secreted protein, extracellular-vesicle-associated material, or assay-specific species. Prodomain processing, alternative splicing, maturation, trafficking, and antibody specificity also influence how ADAM12 is detected. Extracellular-vesicle and exosome-related studies of ADAM12 should follow accepted EV-reporting standards because vesicle preparations can be heterogeneous and functional attribution requires rigorous characterization [41]. Whenever possible, ADAM12-L and ADAM12-S should be distinguished, and ADAM12 abundance should be analyzed together with cellular source, subcellular localization, stromal context, sample type, and downstream biological output.

### Cell-of-origin contexts and known biological functions

The biological meaning of ADAM12 also depends on cell of origin. Four contexts are particularly relevant for this Review: ADAM12-positive CAF/myCAF states, PDGFRα-positive ADAM12-positive mesenchymal stromal cells, tumor-cell ADAM12, and T-cell ADAM12. These contexts share a common molecule but represent distinct biological settings. ADAM12-positive CAF/myCAF states are central to the stromal-checkpoint framework because they connect TGF-β-high myofibroblastic programming, ECM remodeling, fibroblast-state control, and spatial immune exclusion. The broader PDAC literature shows that CAF compartments include myofibroblastic, inflammatory, antigen-presenting, and other plastic states [42]. IL1-driven JAK/STAT signaling and TGF-β signaling antagonistically shape inflammatory versus myofibroblastic CAF states, providing a useful framework for placing ADAM12-positive CAF/myCAF biology within CAF-state plasticity [43]. In the Li et al. single-cell Perturb-seq and functional validation study, ADAM12 perturbation shifted fibroblasts away from TGF-β-associated myofibroblastic programs, elicited IFN-I-responsive antitumor fibroblast states, and improved T-cell-based immune control in models [18]. This provides direct fibroblast perturbation evidence linking ADAM12-positive CAF/myCAF biology to CAF-mediated immune suppression and stromal remodeling. PDGFRα-positive ADAM12-positive stromal cells define a related but distinct mesenchymal niche linked to macrophage efferocytosis, vascular dysfunction, hypoxia/acidosis, CAF-state maintenance, and effector-cell exclusion in preclinical systems [44].

Tumor-cell ADAM12 represents a separate mechanistic category. Earlier work showed that tumor-cell-derived ADAM12 can accelerate breast tumor progression, whereas TGF-β-associated stromal signaling can induce ADAM12 expression in tumor cells, supporting a tumor-stroma feedback model [45, 46]. Additional tumor-cell studies linked ADAM12 to EMT, proliferation, migration, invasion, and EGFR/ERK signaling in pituitary adenoma models [47]. Pharmacologic work in pituitary adenoma cells further connected reduced viability, migration, and invasion to modulation of the ADAM12/EGFR signaling axis, supporting EGFR-linked tumor-cell ADAM12 biology in that disease context [48]. In breast cancer, ADAM12 can promote estrogen-independent growth, illustrating a role in hormone-response or endocrine-escape biology [49]. Invasion-focused work links Twist1-induced ADAM12 to invadopodia and focal-adhesion remodeling [50]. In hepatocellular carcinoma, tetraspanin-8 promotes metastasis by increasing ADAM12m expression, providing another example of tumor-cell ADAM12 regulation in an invasion-associated context [51]. In small-cell lung cancer, E2F1-mediated regulation of ADAM12 expression further supports tumor-cell-specific transcriptional control of ADAM12 [52]. ADAM12 has also been linked to HB-EGF/EGFR signaling cascades and EMT-associated cancer progression in pancreatic cancer-related models [53]. Choriocarcinoma data connect ADAM12 silencing to apoptosis and autophagy-related tumor-cell effects, extending the tumor-type-specific repertoire of tumor-cell ADAM12 biology [54]. Together, these studies show that ADAM12 can act within malignant cells through mechanisms that are experimentally distinct from CAF-derived ADAM12 checkpoint activity.

ADAM12 biology is also not limited to fibroblasts and tumor cells. ADAM12 has been reported to function as a T-cell costimulatory molecule that influences Th1 fate in tissue inflammation, indicating that immune-cell ADAM12 may have functions distinct from CAF-derived ADAM12 or tumor-cell ADAM12 [55]. In addition, the pan-cancer analysis by Yang et al. associated ADAM12 with immune/stromal scores, immune checkpoint genes, TMB, MSI, and immunotherapy-related features, whereas Song et al. reported that ADAM12 is enriched in an ECM-high CAF program and is linked to immune-excluded bladder cancer biology [56, 57]. These datasets broaden the relevance of ADAM12 to stromal and immune biology and help prioritize contexts for functional testing.

Taken together, ADAM12 is best introduced as an isoform-aware and cell-of-origin-dependent regulator or marker of tumor, stromal, and immune programs. Its known biological functions include ectodomain shedding, cell-matrix interaction, integrin-associated adhesion, ECM remodeling, growth-factor signaling, tumor-cell invasion, fibroblast-state regulation, and context-dependent immune associations or modulation. These structural and cellular variables provide the basis for interpreting ADAM12-associated signaling and stromal biology across physical, metabolic, and immune contexts.

## The physical pillar: ECM remodeling, mechanotransduction, and therapeutic access

**Fig. 1:**
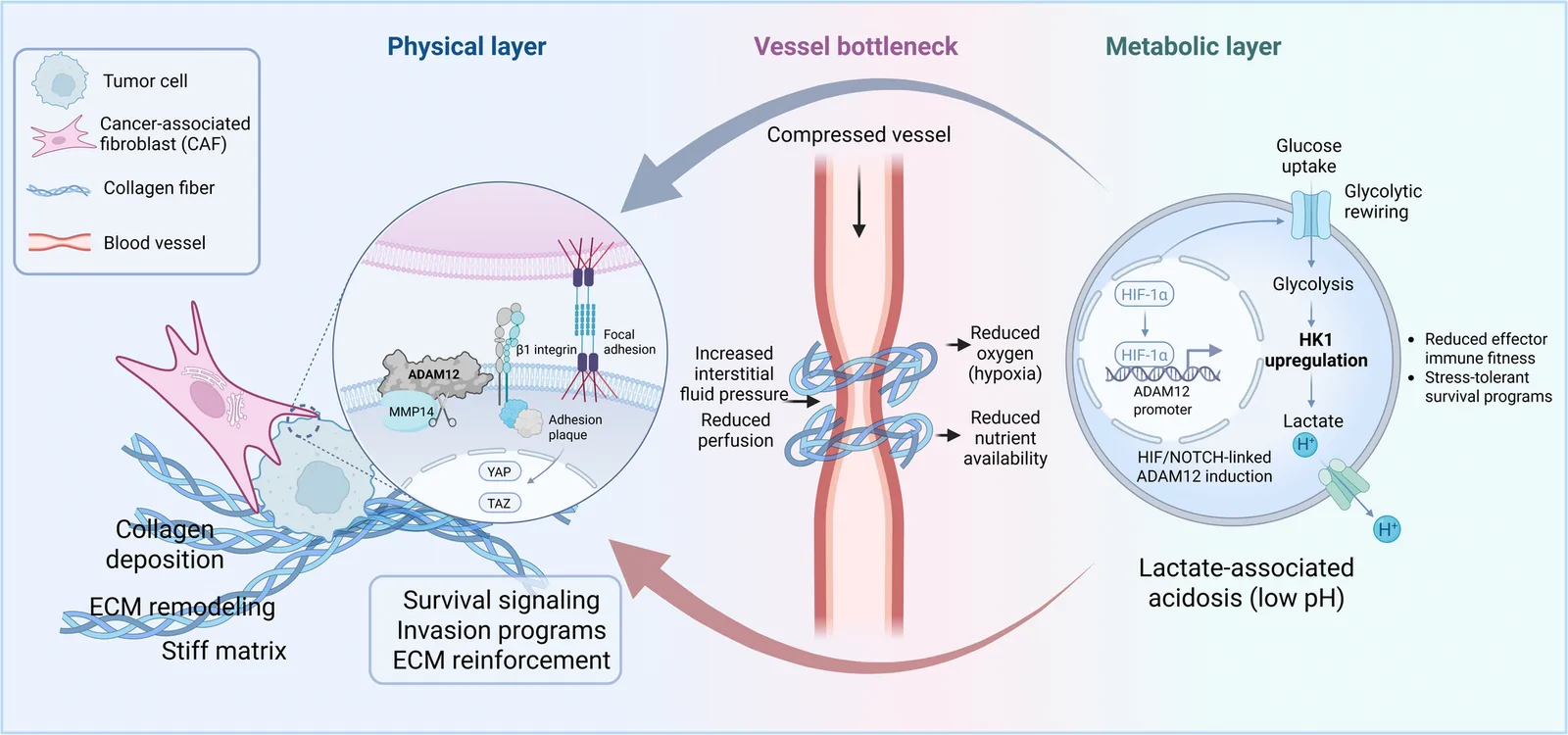
ADAM12-associated stromal remodeling, vessel bottleneck formation, and metabolic stress in the tumor microenvironment. The diagram summarizes three connected layers of ADAM12-associated tumor-stromal barrier biology. In the physical layer, Cancer-associated fibroblasts (CAFs) remodel collagen fibers and Extracellular matrix (ECM), generating collagen deposition, ECM remodeling, and matrix stiffness. The mechanistic inset shows ADAM12 at the cell surface with MMP14, β1 integrin, adhesion plaques, focal adhesion machinery, and YAP/TAZ signaling, linking extracellular remodeling to adhesion-associated mechanotransduction. In the vessel-bottleneck layer, a compressed vessel is shown with increased interstitial fluid pressure, reduced perfusion, reduced oxygen availability, and reduced nutrient availability. In the metabolic layer, the figure depicts HIF-linked ADAM12 induction together with glucose uptake, glycolytic rewiring, HK1 upregulation, lactate generation, lactate-associated acidosis, reduced effector immune fitness, and stress-tolerant survival programs. The figure is intended as a tissue-level schematic of associated stromal, vascular, and metabolic features rather than a claim that ADAM12 alone directly causes each downstream stress phenotype. Figure created with BioRender.com. Abbreviations: ADAM12, A disintegrin and metalloproteinase 12; CAFs, Cancer-associated fibroblasts; ECM, Extracellular matrix; HIF, Hypoxia-inducible factor; HK1, Hexokinase 1; MMP14, Mmatrix metallopeptidase 14; YAP, Yes-associated protein; TAZ, Transcriptional coactivator with PDZ-binding motif

ADAM12-high stromal programs are frequently associated with a physical barrier state (Fig. 1) in which ECM deposition, integrin-linked adhesion signaling, vascular compression, and impaired tissue access converge to support invasion and therapy resistance [58]. ADAM and ADAMTS proteases more broadly participate in pathological tissue remodeling, including ECM turnover, cell migration, proliferation, apoptosis, and remodeling-associated signaling [59]. Within this tissue context, ADAM12 is best treated as a marker or context-dependent organizer of fibrogenic CAF/myCAF and MSC-associated states rather than as a single causal driver of the physical barrier. The physical pillar comprises matrix remodeling, mechanotransduction, and clinically manifest desmoplasia with impaired therapeutic access.

### ADAM12-L, MMP14, and integrin-centered matrix remodeling

Across tumor models, ADAM12 is preferentially enriched in mesenchymal, CAF-like, and fibrogenic states, particularly during TGF-β-associated transitions [60]. This placement suggests that ADAM12 can mark fibrogenic or mesenchymal remodeling states, whereas direct roles in localized proteolysis and invadopodia-associated matrix remodeling are supported mainly in defined tumor-cell systems [46, 61]. In tumor cells, ADAM12 is associated with TGF-β-induced EMT, whereas stromal ADAM12-positive populations should be interpreted separately from tumor-cell ADAM12 biology [46].

One of the strongest mechanistic links between ADAM12 and desmoplastic matrix assembly is its cooperation with integrin- and MMP14-centered remodeling. ADAM12 was identified as a basigin sheddase, and the ADAM12-generated basigin ectodomain can bind β1 integrin and support collagen-related ECM remodeling [62]. In EMT and invadopodia models, ADAM12 colocalizes with ZO-1 in actin- and cortactin-rich structures, where ZO-1 scaffolds ADAM12 with MMP14 to enable localized pericellular matrix degradation [61]. A complementary mechanism is provided by the ADAM12-αVβ3-MMP14 axis, in which cell-surface ADAM12 recruits and conformationally activates MMP14 in a catalysis-independent manner, promoting collagen assembly, matrix rigidification, and apoptosis resistance [63].

This collagen-remodeling module is further shaped by isoform-specific and post-transcriptional regulation. ADAM12-L is the membrane-associated isoform most directly positioned to organize local integrin/MMP14 complexes through its transmembrane and cytoplasmic regions, whereas ADAM12-S is secreted and may influence matrix remodeling through protease-dependent extracellular effects; prodomain splice variants can also differ in processing and trafficking [30]. In invasive pituitary adenomas, both ADAM12-L and ADAM12-S are elevated together with MMP14, whereas silencing studies suggest partially distinct functions, with ADAM12 more closely linked to collagen I degradation and proliferative support and MMP14 contributing more strongly to migration [64]. In breast cancer, the transmembrane and secreted isoforms also show non-identical biological outputs, with ADAM12-S displaying stronger protease-dependent effects on migration and metastasis [65]. ADAM12-L is directly targeted by miR-29 and miR-200 family members, providing a plausible route by which loss of epithelial restraint can amplify matrix-remodeling output [66].

The physical-pillar evidence converges on a core module in which TGF-β-associated ADAM12 induction, integrin engagement, MMP14 recruitment or activation, and collagen/fibronectin-rich matrix remodeling reinforce one another [67]. IGF bioavailability, lipid-context-dependent shedding events, and broad EMT or survival signatures may further modify matrix phenotypes in defined settings [68]. Overall, ADAM12 links fibrogenic state induction with localized proteolysis and matrix assembly, placing it within a matrix-remodeling program that is particularly prominent in collagen-rich, CAF-dense tumors [69].

### Integrin-centered mechanotransduction and stress-responsive signaling loops

Once matrix deposition and remodeling increase local rigidity, mechanical cues are converted into intracellular programs that support adhesion turnover, survival, motility, invasion, and stress tolerance [70]. This conversion is mediated mainly through integrin-based adhesions, which connect the ECM to the actin cytoskeleton and transmit force into biochemical signaling networks, including FAK/Src-centered pathways [71]. In ADAM12-high tumors, ADAM12-associated matrix-remodeling and adhesion programs should be interpreted alongside these canonical stiffness-sensing pathways rather than as proof of a universal ADAM12-driven mechanotransduction mechanism.

ADAM12 may contribute to this process at the interface between extracellular remodeling and adhesion dynamics. As a basigin sheddase, ADAM12 can generate soluble basigin that engages β1 integrin and supports ECM-remodeling programs [62]. In parallel, TGF-β/KAT2A/TAK1 signaling and TGF-β-associated EMT programs can induce or reinforce ADAM12 expression, suggesting a feedback architecture in which mesenchymal state, ECM remodeling, and ADAM12 expression reinforce one another [46, 72]. CAFs cultured on ECM-coated substrates show elevated ADAM12 expression, supporting a model in which ECM engagement strengthens ADAM12-high stromal states [73].

The best-defined intracellular arm is FAK/Src signaling. Integrin-regulated FAK-Src signaling is a canonical route by which rigid ECM promotes motility, invasion, cell-cycle progression, and survival [74]. ADAM12-L provides tumor-cell mechanistic evidence for coupling to this arm: in esophageal cancer, ADAM12-L facilitates focal adhesion turnover and FAK activation, and FAK-JNK-c-Jun signaling in turn sustains ADAM12-L transcription [75]. In breast cancer models, Twist1-induced ADAM12 promotes tumor invasion and metastasis through regulation of invadopodia and focal adhesions [50]. Together with the ADAM12-αVβ3-MMP14 mechanism discussed above, these findings place ADAM12 among the organizers and amplifiers of integrin-centered adhesion signaling [58, 63].

Mechanical signaling also converges on transcriptional plasticity. Rigid matrix, cytoskeletal tension, and focal adhesion signaling are established upstream inputs for YAP/TAZ activity and EMT-associated transcriptional remodeling [67]. Colorectal cancer data further suggest that tumor-cell ADAM12 can promote YAP/TAZ-related output through suppression of Hippo pathway activity [76]. These observations place ADAM12 within matrix-adhesion and EMT-associated signaling rather than establishing a uniform YAP/TAZ mechanism across tumor types.

Stress-responsive growth-factor signaling provides another mechanistic bridge. Under hypoxia, HIF-dependent ADAM12 induction promotes HB-EGF shedding, EGFR activation, and downstream FAK phosphorylation in breast cancer models [77]. NOTCH1 can intensify this route by increasing ADAM12 levels and activity under hypoxia, thereby increasing HB-EGF shedding and invadopodia formation [78]. Expression-based evidence in mucoepidermoid carcinoma also places HIF-1α, NOTCH1, ADAM12, and HB-EGF in the same invasiveness-associated module [79]. The HIF/NOTCH–ADAM12–HB-EGF/EGFR–FAK module therefore provides a model-specific mechanistic link between hypoxic stress and invasion-associated adhesion signaling.

Accordingly, ADAM12 is best interpreted as an amplifier of stiffness-responsive signaling through matrix, integrin, and growth-factor contexts rather than as a single upstream cause of mechanotransduction. The physical pillar therefore does not end with collagen accumulation. It becomes biologically consequential when remodeled ECM is coupled to adhesion turnover, FAK/Src activity, YAP/TAZ-permissive transcriptional states, and stress-responsive EGFR or PI3K/Akt signaling. Matrix stiffness can also reshape tumor-stromal metabolic crosstalk and treatment resistance in contexts such as anti-angiogenic therapy, reinforcing the need to view mechanotransduction and metabolism as coupled tissue-level programs [80]. Translationally, ADAM12-high status can identify an activated mechanochemical niche with potential relevance to drug access, invasion, and stromal adaptation [67].

### Clinical correlates: desmoplasia, interstitial fluid pressure, and therapeutic access

Clinically, the physical pillar is most visible as desmoplasia, high interstitial fluid pressure (IFP), and poor therapeutic access. Dense collagenous stroma compresses vessels, increases diffusion distances, and produces spatially heterogeneous drug exposure, thereby limiting the effectiveness of chemotherapy, biologics, and immune effector cells [81–83]. Independent biophysical studies show that tumor ECM density, stiffness, and architecture can govern nanoparticle diffusion, supporting the broader principle that ECM physical properties directly affect therapeutic distribution [84]. These effects are especially relevant in PDAC and other fibrosis-rich malignancies, where stromal architecture can dominate treatment response even when tumor-cell intrinsic vulnerabilities are present. In this setting, ADAM12 functions as a biomarker and mechanistic participant in a larger desmoplastic program.

ADAM12-associated desmoplasia is supported by several convergent observations. In desmoid fibromatosis, ADAM12 marks mesenchymal fibroblast populations embedded in a highly fibrotic microenvironment, supporting its value as a fibroblast-state marker in a fibrosis-dominant setting [85]. In small intestinal neuroendocrine tumors, tumor-fibroblast crosstalk is linked to ECM programs and mesenteric fibrosis, with ADAM12 detected in this fibrotic context [86]. In glioblastoma, ADAM12 is embedded within matrisome-associated remodeling programs that may influence endothelial behavior and a fibrosis-like niche [87]. In anaplastic thyroid carcinoma and head and neck squamous cell carcinoma, high ADAM12 expression likewise tracks with invasive or ECM-linked stromal behavior [88].

The strongest translational support for the physical barrier model comes from fibrosis-dominant gastrointestinal tumors, especially PDAC. In PDAC and related fibrosis-rich settings, dense desmoplasia can compress vessels, promote hypoperfusion and hypoxia, and restrict delivery of systemic agents [81]. Circulating ADAM12 provides a blood-based stromal-activation readout in PDAC, where elevated or persistent serum levels are associated with poorer outcome and reduced treatment benefit [89]. Preclinical vaccination against ADAM12 reduced CAF abundance and tumor desmoplasia, delayed tumor growth, promoted vascular normalization, alleviated hypoxia, and redistributed CD8 + T cells [90]. These data support ADAM12 as a stromal-state marker and preclinical entry point for interrogating ADAM12-positive fibrogenic compartments.

A similar translational pattern is evident in upper gastrointestinal malignancies, where ADAM12 is best supported as a stromal-state biomarker. In esophageal adenocarcinoma, serum ADAM12 is linked to IL-6-producing CAF activity, stromal fibrosis, and poor response to neoadjuvant therapy [91]. In metastatic esophagogastric adenocarcinoma, high baseline serum ADAM12 is likewise associated with shorter progression-free survival during cytotoxic treatment [92]. Serum biomarker studies in esophageal squamous cell carcinoma and urinary biomarker studies in gastric cancer further support the feasibility of non-tissue ADAM12-related readouts [93, 94]. A more recent urinary protein biomarker panel for gastric cancer also supports the clinical interest in urine-based protein detection [95]. These observations are strengthened by the broader concept that circulating markers of altered matrix turnover can report fibrosis-associated and treatment-relevant stromal states in a liquid-biopsy format [96]. Together, these clinical associations position ADAM12 as a measurable indicator of activated CAF-rich stroma and barrier-associated disease biology.

For the physical barrier question, the strongest studies connect ADAM12 to CAF activation, ECM accumulation, vascular normalization or dysfunction, hypoperfusion, hypoxia, IFP-related access barriers, and reduced therapeutic benefit. Studies centered on stemness signatures, methylation status, pan-cancer prognosis, or general immune-cold phenotypes remain relevant elsewhere in the Review but are less directly informative for desmoplastic access barriers. This evidence hierarchy keeps the physical pillar focused on matrix, perfusion, and tissue-delivery biology.

The available evidence supports a clinically relevant triad of desmoplasia, elevated IFP, and impaired therapeutic access. Within this stromal-checkpoint framework, ADAM12 is best treated as a biomarker and, in preclinical settings, a functional marker of ADAM12-positive fibrogenic compartments associated with collagen-rich remodeling, abnormal vasculature, poor perfusion, hypoxia, and immune exclusion. This view is reinforced by the broader literature on fibrosis-associated biomarkers, which increasingly treats matrix-remodeling signals as measurable surrogates of disease-driving stromal pathology across cancer and chronic tissue injury [96]. The therapeutic implication is selective stromal normalization: ADAM12-informed strategies should identify tumors in which CAF-rich physical barriers dominate treatment failure and may be vulnerable to approaches that decompress vessels, lower IFP, improve intratumoral drug delivery, and weaken the structural basis of therapeutic resistance [97, 98].

## The metabolic pillar: hypoxic stress, metabolic coupling, and ADAM12-linked stromal adaptation

Hypoxia is an active stress program in stromal-rich tumors. Hypoperfusion, matrix compression, glycolytic coupling, lactate accumulation, acidosis, immune suppression, and treatment resistance can reinforce one another as tissue-level stress programs [99]. ADAM12 intersects with this biology as a responder, marker, and amplifier of hypoxia-adapted tumor-stromal states. Direct perturbation evidence supports HIF-linked ADAM12 induction in defined breast cancer models [77]. A related Notch-linked feedback module connects hypoxia, NOTCH1-dependent ADAM12 activity, HB-EGF shedding, and EGFR/FAK-associated invasive signaling [78]. Stromal evidence separately supports ADAM12-positive PDGFRα-positive MSC/CAF compartments as contributors to pathological angiogenesis, hypoxia, acidosis, and immunosuppressive niche maintenance in preclinical systems. These mechanisms connect model-specific tumor-cell signaling with broader ADAM12-high associations in hypoxia, glycolysis, lactate biology, and therapy resistance.

### Hypoxia-responsive ADAM12 induction and notch-linked feedback

Hypoxia activates HIF-dependent transcriptional programs that enable cancer and stromal cells to adapt to oxygen and nutrient limitation [100]. In breast cancer, HIF-dependent ADAM12 induction promotes HB-EGF shedding, EGFR activation, downstream FAK phosphorylation, migration, invasion, and lung metastasis, and inhibition of ADAM12 expression or activity attenuates these hypoxia-induced phenotypes [77]. This provides direct functional evidence for a hypoxia–ADAM12–HB-EGF/EGFR–FAK axis in that model. In adenoid cystic carcinoma, HIF-1α, ADAM12, NOTCH1, and HB-EGF are coordinately upregulated in association with invadopodia formation and invasiveness, extending the same expression module to another invasive tumor setting [101].

Hypoxia can also regulate ADAM12 through Notch-linked signaling crosstalk. In tumor cells, hypoxia activates NOTCH signaling, and NOTCH1-dependent ADAM12 upregulation increases ADAM12 proteolytic activity, HB-EGF shedding, EGFR activation, downstream FAK phosphorylation, and invadopodia formation [78]. This mechanism is best conceptualized as a two-arm feedback loop. In the upstream arm, hypoxic stress and cell-contact-dependent NOTCH activation converge to increase ADAM12 abundance or activity. In the downstream arm, ADAM12-mediated HB-EGF shedding activates EGFR/FAK signaling in neighboring or adjacent tumor cells, thereby promoting invadopodia formation and invasive behavior. This pathway links hypoxia sensing, Notch signaling, ADAM12 sheddase activity, and EGFR/FAK output. Evidence from ameloblastoma and adenoid cystic carcinoma further places NOTCH1, HIF-1α, ADAM12, and HB-EGF within a shared invasiveness-associated module [60, 101]. At the systems level, this interpretation is consistent with the broader role of HIF signaling in coordinating glycolysis, survival signaling, and resistance programs in low-oxygen tumors [102].

Hypoxia also rewires cellular metabolism. HIF-driven adaptation promotes glycolytic flux, limits pyruvate entry into mitochondrial oxidation, and reshapes the balance between glycolysis and OXPHOS in a manner that favors survival in poorly perfused tumors [103]. IDH2-dependent stabilization of HIF-1α can also promote metabolic reprogramming and chemoresistance in urothelial cancer, illustrating how HIF-centered metabolic resistance can arise through multiple molecular routes [104]. Under these conditions, lactate accumulates as both a metabolic by-product and a signaling metabolite, contributing to macrophage polarization, immune suppression, and niche stabilization [105]. Lactate may also participate in epigenetic regulation through lactylation, providing a broader conceptual link between metabolic stress and gene-regulatory persistence [106]. Epigenetic and post-transcriptional mechanisms may further reinforce ADAM12 expression. Taken together, hypoxia emerges as an upstream inducer and amplifier of ADAM12 through HIF-dependent, Notch-linked, and stress-responsive mechanisms. The Notch arm is particularly important because it provides a defined feedback route from hypoxic cell-contact signaling to ADAM12-dependent HB-EGF shedding and EGFR/FAK-driven invasion.

### ADAM12-linked metabolic effectors and stromal metabolic coupling

ADAM12 intersects with hypoxia-linked metabolic adaptation at multiple evidence levels: tumor-cell studies link ADAM12 to glycolytic or PI3K-Akt-associated effectors, while stromal studies connect ADAM12-positive MSC/CAF states to hypoxia, acidosis, pathological angiogenesis, and immunosuppressive niche maintenance. CAF metabolic plasticity is governed by tissue context and by interactions among matrix, vasculature, nutrient availability, and immune pressure. In the reverse Warburg framework, stromal fibroblasts can adopt aerobic glycolysis, produce lactate and other energy-rich metabolites, and support adjacent cancer cells through metabolic coupling [107, 108]. More broadly, metabolic exchanges between tumor and stromal compartments help maintain growth under fluctuating oxygen and nutrient availability, placing cancer metabolism within the surrounding TME [109, 110].

Within this broader framework, ADAM12 interfaces with metabolic effector pathways. In small cell lung cancer, ADAM12-S increases HK1 expression and glycolytic flux, and ADAM12-S manipulation alters proliferation, colony formation, migration, and invasion [111]. In non-Hodgkin lymphoma, ADAM12 promotes proliferation, adhesion, PI3K-Akt/GSK-3β signaling, and cell adhesion-mediated drug resistance, supporting a link between ADAM12 and therapy-tolerant metabolic or survival signaling [112]. In breast cancer, ADAM12-L has been linked to PI3K/Akt-associated drug tolerance and invasive behavior. These tumor-cell studies support model-specific links between ADAM12, glycolytic output, nutrient-stress-associated effectors, and therapy tolerance.

ADAM12 may connect stromal remodeling to metabolic adaptation through proteolysis-dependent growth-factor signaling, but the evidence remains indirect outside defined tumor-cell settings. FSTL3-ADAM12 interactions link ADAM12 to growth-factor pathways that may intersect with PI3K-Akt-mTOR nutrient sensing [113]. Stromal-cell depletion studies provide stronger tissue-level support for links among ADAM12-positive compartments, vascular dysfunction, hypoxia, acidosis, and immune features [44]. These data support contextual coupling between ADAM12-positive stroma and metabolic stress rather than a direct claim that ADAM12 initiates metabolic coupling.

Overall, current evidence supports ADAM12 as an amplifier and biomarker of tumor-stromal metabolic adaptation. ADAM12 has direct functional links to HK1, PI3K-Akt-associated survival signaling, and drug resistance in tumor-cell models; ADAM12-positive stromal compartments are functionally associated with vascular dysfunction, hypoxia, acidosis, and CAF-state maintenance in preclinical systems; and broader links to reverse Warburg metabolism, lactate biology, and nutrient sensing provide tissue-level context for the metabolic pillar.

### Lactate, acidosis, and the ADAM12-linked stromal niche

A major consequence of glycolytic adaptation in tumor-stromal niches is extracellular lactate accumulation and acidosis. These features are not inert by-products; they can shape invasion, matrix remodeling, immune-cell function, and therapeutic resistance in the TME [114]. In solid tumors, extracellular pH can fall below physiologic levels, and this acidic milieu may support protease activity, ECM remodeling, and reduced immune fitness across multiple immune compartments, not only CD8⁺ T cells [115]. Thus, the metabolic pillar feeds back into the physical and immune pillars of stromal resistance.

Recent work has made the stromal contribution more concrete. Glycolytic pCAFs can create a high-lactate microenvironment that promotes aggressive pancreatic cancer behavior through lactate-driven histone lactylation and perineural invasion programs [116]. Tumor-derived lactate can also activate CAFs directly, inducing transcriptional reprogramming that strengthens IL-8-mediated macrophage recruitment and immunosuppressive remodeling in lung cancer models [117]. Together, these findings support a bidirectional model in which tumor glycolysis activates fibroblasts and fibroblast glycolysis reinforces lactate accumulation.

ADAM12 intersects with this niche most convincingly through ADAM12-positive stromal compartments. Genetic depletion of ADAM12-positive PDGFRα-positive mesenchymal stromal cells restores functional vasculature, reduces hypoxia and acidosis, normalizes CAF states, and improves antitumor immunity [44]. In preclinical PDAC, targeting ADAM12-positive fibrogenic cells reduces desmoplasia, promotes vascular normalization, and alleviates hypoxia [90]. Together with fibroblast-checkpoint evidence, these data position ADAM12 as a candidate stabilizer of hostile stromal niches rather than a universal cause of hypoxia or acidosis.

Lactate and acidosis are central components of poorly perfused, hypoxic TMEs. Lactate can polarize macrophages toward VEGF-expressing M2-like states, suppress cytotoxic immune activity, and reinforce tumor-supportive niches [118]. Lactate also contributes to immune-cell dysfunction, although its effects vary by concentration, cell type, nutrient state, and tissue context. In melanoma, tumor-derived lactate reduces intratumoral ILC2 abundance and weakens the IL-33/ILC2/eosinophil axis, whereas reduced lactate production restores ILC2-dependent antitumor immunity [119]. Lactate can also increase CD8⁺ T-cell stemness and augment antitumor immunity in selected experimental settings [120]. Recent work shows that MCT11-mediated lactate uptake can enforce exhausted T-cell dysfunction, making lactate transport and immune-cell metabolic state central variables in interpreting metabolic immune fitness [121]. These findings show how metabolic stress can shape tissue-resident, exhausted, and type 2 immune programs in stromal-rich tumors; in this Review, they are used as contextual biology for interpreting ADAM12-high stromal states rather than as ADAM12-specific mechanisms.

Overall, ADAM12 marks, and in selected models may reinforce, hypoxia-associated stromal adaptation; however, direct links to lactate accumulation and acidosis remain largely tissue-level or compartment-level associations. Direct evidence supports HIF- or Notch-linked ADAM12 induction and ADAM12-dependent growth-factor signaling in tumor-cell models. Stromal perturbation studies separately support ADAM12-positive MSC/CAF compartments as contributors to vascular dysfunction, hypoxia, acidosis, CAF-state maintenance, and immune suppression in preclinical systems. Broader links among lactate accumulation, histone lactylation, macrophage recruitment, ILC2/eosinophil biology, and therapy resistance extend the metabolic pillar into a stromal-barrier program, but they should not be read as uniformly ADAM12-dependent.

## The immune pillar: tissue-level immune geography, suppression, and immune-state remodeling

**Fig. 2:**
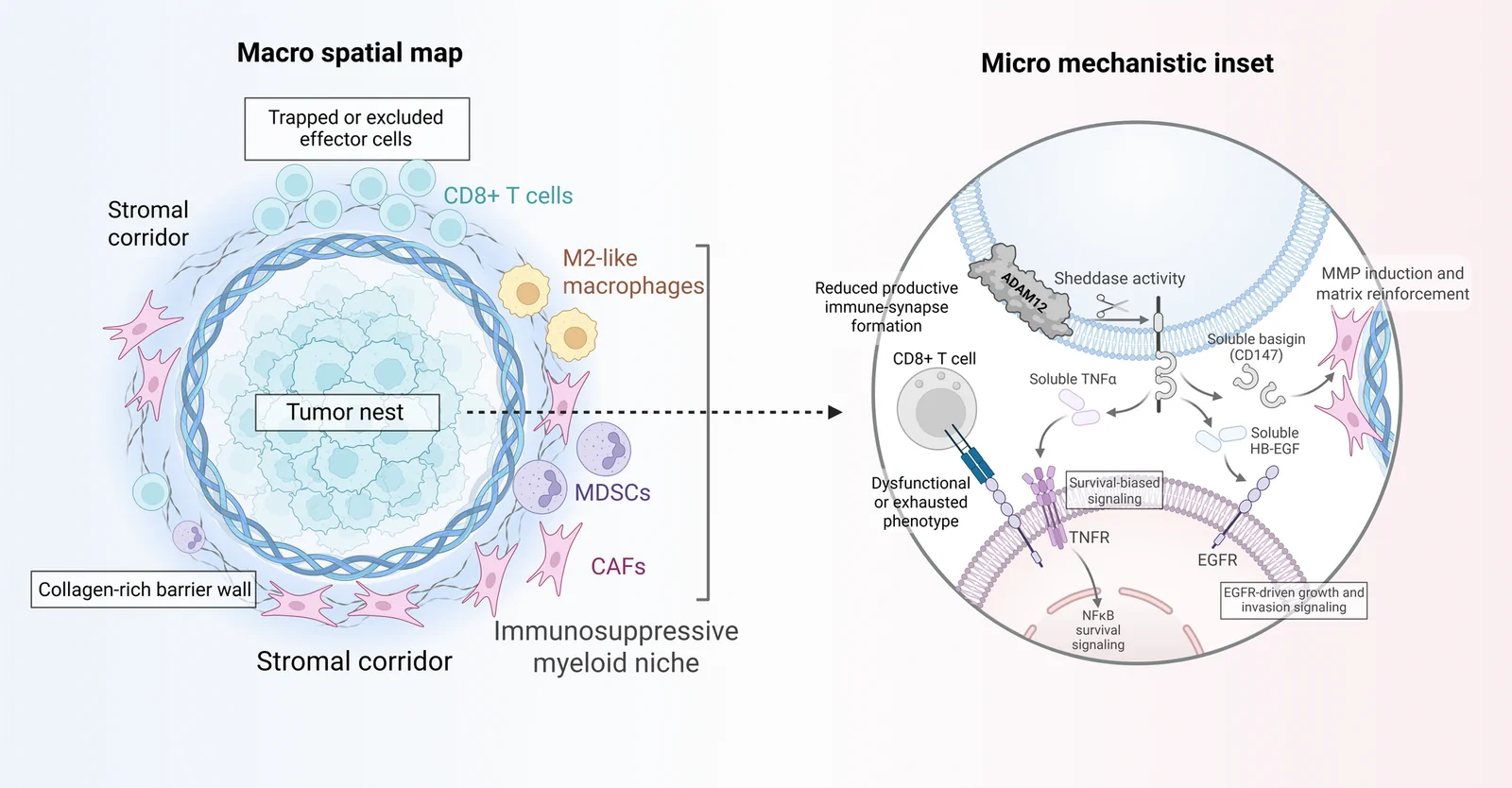
CAF-associated immune-excluded spatial organization and context-specific ADAM12-linked sheddase/paracrine remodeling. The figure summarizes a macro spatial map and micro mechanistic inset for ADAM12-high stromal niches. The left panel shows a tumor nest surrounded by a collagen-rich barrier wall and CAF-lined stromal corridors. CD8 + T cells are shown as trapped or excluded effector cells, whereas MDSCs, M2-like macrophages, and CAFs occupy an immunosuppressive myeloid stromal niche. The right panel depicts membrane-associated ADAM12 with sheddase activity and selected soluble or paracrine outputs, including soluble basigin (CD147), soluble HB-EGF, and a soluble TNFα/TNFα–NF-κB axis that should be interpreted as context-specific rather than as a pan-cancer ADAM12 output. These cues are linked in the schematic to TNFR/NF-κB survival signaling, EGFR-driven growth and invasion signaling, reduced productive immune-synapse formation, dysfunctional or exhausted phenotype, and MMP induction and matrix reinforcement. Figure created with BioRender.com. Abbreviations: ADAM12, A disintegrin and metalloproteinase 12; CAFs, Cancer-associated fibroblasts; CD147, basigin; CD8⁺ T cells, Cytotoxic T lymphocytes; EGFR, Epidermal growth factor receptor; HB-EGF, Heparin-binding EGF-like growth factor; MDSCs, Myeloid-derived suppressor cells; MMP, Matrix metalloproteinase; NF-κB, Nuclear factor kappa B; TNFα, Tumor necrosis factor alpha; TNFR, Tumor necrosis factor receptor

A recurring immune pattern in ADAM12-enriched stromal contexts is ineffective immune positioning, in which effector lymphocytes, suppressive myeloid populations, fibroblast-rich matrix corridors, lymphatic vessels, and tissue-resident immune programs can become spatially organized in ways that limit productive antitumor immunity [122]. This architecture can create a functional immune-cold phenotype even when immune cells or checkpoint molecules are detectable, because immune abundance without appropriate localization, antigen presentation, and effector access rarely translates into durable antitumor control [123]. Lymphatic drainage and TDLN priming provide tissue-level determinants of antitumor immunity and therapeutic response [124]. Metabolic immune fitness also shapes solid-tumor immunotherapy responses [25]. The study by Guo et al. illustrates this principle in solid-tumor immunotherapy models: terminally exhausted CD8 + tumor-infiltrating lymphocytes can retain cytotoxic effector potential, and IL-10-driven metabolic reprogramming through oxidative phosphorylation can enhance their function and improve antitumor immunotherapy responses [25]. Tumor-type-specific immune-evasion reviews in hepatocellular carcinoma further illustrate how abnormal angiogenesis, chronic inflammation, ECM remodeling, metabolic stress, and suppressive immune-cell circuits jointly determine immunotherapy responsiveness [125]. Immune escape in this setting is a tissue-level process composed of spatial exclusion, CAF/MSC-driven immune suppression, lymphatic and TDLN-associated immune trafficking, metabolic immune fitness, sheddase-linked rewiring of surface and paracrine cues, and regulatory programs that stabilize the resulting cold-tumor state. ILC2/eosinophil biology and type 2 immunity represent tissue-level immune axes that shape this stromal geography [126]. Together, these features provide the tissue-level background for the CAF-associated immune-exclusion and sheddase/paracrine remodeling architecture shown in Fig. 2, in which lymphatic/TDLN biology and ILC2/type 2 immune contexts remain modifiers of immune geography rather than ADAM12-specific direct mechanisms.

### Spatial exclusion, lymphatic/TDLN biology, and tissue-level immune compartmentalization

Immune-excluded tumors are now recognized as a distinct state that differs from both inflamed and desert phenotypes [123]. In this state, T cells are not fully absent, but are spatially restricted to stromal regions and remain unable to penetrate malignant nests efficiently [122]. Foundational TGF-β studies provide a mechanistic precedent for this architecture, showing that stromal TGF-β signaling can contribute to T-cell exclusion and reduced PD-L1 blockade response, and that TGF-β-driven immune evasion can prevent acquisition of TH1-effector phenotypes in metastatic colon cancer models [20, 21]. Productive antitumor immunity also depends on dendritic-cell trafficking, antigen transport, lymphatic drainage, and TDLN priming [124]. It also depends on B-cell/TLS-like organization, tissue-resident lymphoid populations, type 2 immune context, and the balance among TAMs, MDSCs, Tregs, neutrophils, eosinophils, and fibroblast-associated immune niches [126]. Such compartmentalization is especially relevant in fibroblast-rich tumors, where ECM density, TGF-β-driven myofibroblastic programs, vascular or lymphatic dysfunction, and suppressive myeloid crosstalk cooperate to block productive immune engagement [127]. CAFs are increasingly viewed as active gatekeepers of antitumor immunity, with subtype-specific functions that may be suppressive, permissive, or restraining depending on context [128].

Within this framework, ADAM12 provides direct cell-context-specific evidence for immune-state remodeling. In the T11 TNBC model, cancer-cell Adam12 ablation increases intratumoral B-cell and T-cell abundance, reduces neutrophil and myeloid-derived suppressor cell enrichment, and sensitizes tumors to combined PD-1 and CTLA-4 blockade, although acquired resistance can still emerge [129]. The most direct CAF-centered evidence comes from Li et al., who identified ADAM12 as a fibroblast checkpoint that links TGF-β-associated myofibroblastic programming to suppression of IFN-I-responsive antitumor fibroblast states and limited T-cell-based immune control [18]. In that framework, ADAM12-positive CAF/myCAF biology is relevant not only because it marks fibrotic stroma, but because ADAM12 perturbation can reconfigure fibroblast-state structure and may relieve a fibroblast-mediated barrier to antitumor immunity. Other CAF-mediated immune-evasion mechanisms reinforce the same conceptual point; for example, CAF-derived IGF2 can drive fibroblast-mediated tumor immunoevasion and resistance to immunotherapy through IGF1R/PI3K-AKT signaling in models [130]. In parallel, depletion of slow-cycling PDGFRα-positive ADAM12-positive mesenchymal stromal cells restores functional tumor vasculature, reduces hypoxia and acidosis, normalizes CAF states, restricts macrophage efferocytosis, and increases effector T-cell infiltration [44]. These studies support ADAM12-positive tumor, CAF, and MSC compartments as contributors to immune exclusion in selected preclinical contexts.

These functional observations are consistent with a broader spatial logic described across solid tumors. Fibroblast-rich immune exclusion is often reinforced by reciprocal interactions between matrix-producing stromal cells and suppressive myeloid populations, creating desmoplastic niches that are poorly penetrated by effector cells and poorly connected to productive antigen-presentation circuits. Spatial transcriptomic studies have shown that fibroblast- and macrophage-enriched structures can organize immune-excluded architecture and predict poor benefit from checkpoint therapy [131]. More generally, modern spatial profiling indicates that immune localization within tumors is at least as informative as immune abundance, because stromal trapping can uncouple infiltration from function [122]. Spatial technologies now complement abundance-based biomarkers by resolving tumor immune ecosystems in situ [132].

Lymphatic vessels add a distinct regulatory layer to this tissue geography. Lymphatic drainage and TDLN priming shape antigen transport, dendritic-cell trafficking, metastatic seeding, immune-cell localization, and response to therapy [133]. Recent synthesis of lymphatic biology in cancer immunotherapy further emphasizes that lymphatic vessels actively regulate tissue immunity and can influence both antitumor immune priming and immunotherapy response [133]. Direct immunotherapy studies also show that the PD-1/PD-L1 checkpoint can restrain T-cell immunity within tumor-draining lymph nodes, underscoring the immunoregulatory function of TDLNs [134]. Skin-cancer studies emphasize that lymphatic vessels coordinate local inflammation and immunity and may influence prognosis and therapeutic response, reinforcing the need to integrate lymphatic biology into stromal-checkpoint models [135]. ADAM12-high stroma is therefore discussed within the larger lymphatic/TDLN immune geography that shapes stromal resistance.

ADAM12-high states recur in immune-hostile tumor contexts, although most human data remain correlative. In LUAD, ADAM12 correlates with unfavorable immune markers and inferior outcome [136]. In ccRCC, high ADAM12 abundance associates with PD-1, PD-L1, CTLA-4, and an immunosuppressive infiltrate profile [137]. Bladder cancer and pan-cancer datasets link ADAM12 with ECM-high CAF programs, immune/stromal scores, immune checkpoint genes, TMB, MSI, and immunotherapy-related features [56, 57]. Productive immune structures such as B-cell/TLS organization should be analyzed alongside ADAM12-high immune-excluded states because they can promote immunotherapy response [138]. The strongest interpretation is that ADAM12 marks immune-hostile stromal organization in several datasets, whereas functional exclusion must be established in model-specific perturbation systems.

The immune-excluded phenotype is also sustained by regulatory circuits that converge on invasive, matrix-remodeled, and fibroblast-supported tumor states. In bladder cancer, the super-enhancer-associated lncRNA SNHG15 recruits FOSL1 to activate ADAM12 transcription and reinforce WNT-linked malignant progression [139]. In gastric cancer, ADAM12 has been connected to Th2-skewed and mast-cell-enriched immune contexts, and additional gastric datasets support an association between ADAM12-containing TME signatures and altered immune composition [118, 140]. Type 2 immunity is context-sensitive: ILC2-eosinophil programs, tissue-resident immune cells, and lactate-sensitive immune circuits can restrain or support tumor growth according to tissue context, tumor stage, and metabolic state [119]. A related ILC2-focused melanoma study further supports the idea that ILC2-derived GM-CSF can recruit and activate eosinophils and that PD-1 blockade can unleash ILC2/eosinophil-dependent antitumor immunity in selected settings [141]. ADAM12-associated Th2 or mast-cell signals therefore fit into a broader tissue-level immune-state shift.

These findings also help explain why stromal normalization may complement checkpoint blockade. Interventions directed against fibroblast programs can improve CD8⁺ T-cell activation, alter macrophage polarization, and loosen the spatial barrier that prevents effective tumor-immune contact [142]. More broadly, CAF-focused therapeutic strategies are increasingly being developed to reverse immune exclusion and support renewed sensitivity to immunotherapy, with patient selection remaining essential because CAF biology is heterogeneous and some stromal programs may restrain tumor progression [143]. ADAM12 itself also has immune-cell biology outside CAFs: in tissue inflammation, ADAM12 has been reported to function as a T-cell costimulatory molecule influencing Th1 fate, indicating that cell-of-origin must be considered when discussing ADAM12 immunomodulation [55]. Thus, ADAM12-associated spatial exclusion is a layered stromal and tissue-level barrier in which matrix organization, CAF or MSC state, vascular and lymphatic access, antigen-presentation geography, suppressive myeloid co-localization, and tissue-resident immune programs jointly reduce the probability of productive antitumor immunity.

### Sheddase-linked paracrine rewiring and immune-state modulation

Spatial exclusion alone does not fully explain the immune barrier. Beyond shaping tissue architecture, ADAM12 can influence immune-relevant communication through proteolytic release of selected ligands and altered availability of surface-associated signaling molecules, a process captured here as sheddase-linked paracrine rewiring [144]. In tumors, ectodomain shedding and related protease activity can reshape gradients of growth factors, cytokines, receptor ligands, and adhesion cues, thereby influencing immune trafficking, stromal activation, endothelial behavior, and resistance states [145]. This is particularly relevant in fibroblast-rich tumors, where communication among CAFs, myeloid cells, endothelial or lymphatic compartments, antigen-presenting cells, and malignant cells is a major determinant of immune suppression and therapy response [146]. Immune-checkpoint-related tumor-cell programs may also intersect with ADAM12-containing gene networks, as shown by HLA-G neo-expression studies in clear cell renal carcinoma cell lines [147]. For ADAM12 specifically, direct sheddase substrates should be distinguished from broader CAF-paracrine, immune-checkpoint-associated, or ADAM-family mechanisms.

A central ADAM12-linked signaling route is HB-EGF release and downstream EGFR pathway activation. The hypoxia-induced ADAM12-HB-EGF/EGFR/FAK axis may have immune-context relevance because growth-factor activation can coexist with immune-hostile stromal organization, although direct immune regulation by this axis remains model-dependent. Additional work on soluble basigin and TNF-α illustrates how ADAM12-linked sheddase biology can modify paracrine signaling in selected contexts, with TNF-α–NF-κB best treated as a glioblastoma temozolomide-resistance context rather than a pan-cancer ADAM12 mechanism [62, 148].

CAF-immune crosstalk extends this effect at the microenvironmental level. CAFs use cytokines, chemokines, growth factors, extracellular vesicles, and matrix-derived cues to suppress cytotoxic immunity, alter dendritic-cell and antigen-presentation circuits, and recruit or sustain suppressive immune populations [149]. These programs frequently converge on TAMs, MDSCs, Tregs, neutrophils, and other myeloid compartments that amplify checkpoint resistance, but they may also affect B-cell/TLS organization, tissue-resident lymphoid cells, and lymphatic or TDLN-associated immune trafficking [114]. ADAM12-positive CAF or MSC states fit within this broader CAF-immune framework because they have been functionally linked to myofibroblastic state maintenance, vascular dysfunction, hypoxia/acidosis, macrophage efferocytosis, and impaired effector-cell infiltration in models. In cholangiocarcinoma and other fibrosis-rich tumors, fibrogenic and myeloid communication circuits dominate the immunosuppressive TME and limit cytotoxic surveillance [150].

ADAM12 surface availability is itself dynamically regulated, which adds another layer to its signaling output. Membrane-associated ADAM12-L undergoes constitutive clathrin- and Grb2-dependent internalization, and this trafficking behavior regulates cell-surface dynamics and substrate processing [33]. Related work further supports the idea that ADAM12 function is controlled not only at the transcriptional level but also by maturation, trafficking, isoform composition, and post-translational regulation that determine substrate exposure and signaling magnitude [30, 38]. In the TME, the immunological impact of any ADAM12-linked signal depends on cell type, isoform, subcellular location, and timing of protease activity.

Upstream control of ADAM12 links it to stress-adaptive and suppressive signaling networks. Twist1 induces ADAM12 within an invasive EMT-associated program that regulates invadopodia and focal adhesions [50]. JMJD2A can transactivate ADAM12 and support proliferative output in selected cancer-cell contexts [151]. Smad3-centered circuits can promote macrophage-to-myofibroblast transition and expand fibrogenic CAF populations that contribute to immune-suppressive stroma [152]. These mechanisms place ADAM12 near EMT, chromatin regulation, and fibroinflammatory signaling without implying a single shared regulatory hierarchy.

This interpretation is reinforced by general work on ADAM proteases in cancer. Multiple ADAM family members can contribute to immune evasion by reshaping membrane-bound signaling cues, growth factor availability, cytokine or receptor-ligand exposure, and stromal-tumor communication, while also presenting substantial therapeutic challenges because of substrate diversity, tissue context, and overlapping family functions [144]. For ADAM12 specifically, proteolytic and cell-surface functions have been linked to ligand release, adhesion, EGFR/ERK, FAK, and stromal-state programs that can favor immunosuppressive tissue organization in selected contexts. Direct ADAM12-dependent immune substrates remain less completely mapped than its roles in HB-EGF shedding, basigin shedding, integrin/MMP14-centered remodeling, and fibroblast-state regulation.

Therapeutically, this makes the ADAM12 axis attractive but still evidence-dependent. Triptolide suppresses pituitary adenoma cell viability, migration, and invasion in part by attenuating ADAM12/EGFR signaling [48], and ADAM12 silencing can promote apoptosis and autophagy-related effects in choriocarcinoma cells [54]. These studies support druggable ADAM12-linked signaling. Immune-oriented support also comes from the T11 TNBC model, in which cancer-cell Adam12 ablation altered immune infiltration and improved response to checkpoint blockade [129]. More broadly, CAF-targeting and CAF-reprogramming strategies are entering translational development as ways to overcome immunotherapy resistance by disrupting stromal-immune crosstalk alongside tumor-cell-directed therapy [153]. The therapeutic implication is isoform-aware and cell-of-origin-aware stromal priming in tumors where ADAM12-positive CAF, MSC, or tumor-cell programs sustain immunosuppressive signaling.

### Regulatory lock-in of ADAM12-high immune-cold states

Once established, an immune-cold state is rarely transient. It is more often maintained by interacting regulatory layers that stabilize stromal architecture, suppressive immune geography, metabolic stress, and impaired immune re-entry [154]. In this context, some ADAM12-high tumors display a persistent barrier phenotype sustained by transcriptional, post-transcriptional, epigenetic, and tissue-level reinforcement. This framing is more useful than treating every ADAM12-associated ncRNA as an isolated immune mechanism. The key issue is persistence of an ADAM12-linked stromal or tumor-cell program that can coexist with CAF-rich matrix corridors, suppressive myeloid niches, altered antigen-presentation circuits, lymphatic or TDLN-related immune trafficking, and non-classical tissue-resident immune pathways.

Non-coding RNA circuits provide an additional layer of ADAM12 regulation. In TNBC, miR-30b has been reported to regulate ADAM12-associated proliferation, migration, and invasion phenotypes [155]. In glioblastoma, ADAR2 reduction and miR-135b-mediated regulation intersect with ADAM12-containing programs linked to anchorage-independent growth, proliferation, migration, self-renewal, and infiltration [156]. These examples support RNA-level regulation of ADAM12-linked invasive or mesenchymal programs, while their contribution to immune-cold tissue architecture remains context-dependent.

Epigenetic fixation adds a further layer of stability. In uveal melanoma, ADAM12 promoter hypomethylation is associated with early metastasis and ADAM12 upregulation [157]. Epigenetically supported ADAM12 expression may help stabilize invasive, mesenchymal, or stromal-interacting states over time. More broadly, immune escape commonly depends on genetic, epigenetic, and post-transcriptional changes that lock tumors into camouflage, coercion, and cytoprotection states, while epigenetic modulation can also influence antigen presentation and immune-cell function [158, 159]. ADAM12 therefore belongs within a durable regulatory-state framework.

Epitranscriptomic and metabolic-to-epigenetic regulation may contribute to regulatory persistence. m6A methylation regulates therapy resistance, cell-state plasticity, and oncogenic persistence [160]. Lactate-associated chromatin remodeling and histone lactylation can influence macrophage polarization, fibroblast activation, invasion, and treatment resistance [116]. These processes may coexist with ADAM12-high states in lactate- and acidosis-rich niches, but direct ADAM12 control should not be inferred without perturbation evidence.

Circulating ADAM12 may provide a partial clinical readout of this stabilized state as a biomarker of stromal activation or aggressive disease biology. In colorectal cancer, serum-based ADAM12 measurements have been associated with stromal activation and poor prognosis [161]. In PDAC, circulating ADAM12 functions as a blood-borne proxy for stromal activation and correlates with treatment benefit and outcome [89]. These data support ADAM12 as a measurable surrogate of persistent barrier biology, especially when integrated with spatial immune profiling, CAF-state assessment, perfusion or hypoxia measures, and lymphatic or TDLN-related readouts.

ADAM12-high states also intersect with therapy-resistance programs. In breast cancer, ADAM12 isoforms have been linked to estrogen-independent growth, and ADAM12-L has been linked to PI3K/Akt-associated acquired 5-fluorouracil resistance [162]. Endocrine-resistance literature places ADAM-family members within broader growth-factor and stromal compensation networks [163]. Hypoxia-metabolism circuits such as IDH2-HIF-1α signaling can reinforce chemotherapy resistance and low-immunity phenotypes [104]. These examples support a composite view of immune-cold states built from fibroblast activation, metabolic stress, vascular or lymphatic abnormalities, defective antigen presentation, suppressive myeloid programs, and persistent regulatory signaling.

The ADAM12-associated immune-cold phenotype should be treated as a multilayered outcome rather than a single pathway. Functional evidence supports ADAM12-dependent remodeling of tumor-cell or fibroblast states in selected models, whereas mechanistic and correlative evidence links ADAM12-high programs to sheddase signaling, EMT, ncRNA regulation, stromal activation, and circulating biomarker readouts. Broader immune-cold architecture includes lymphatic drainage, TDLN priming, dendritic-cell trafficking, antigen transport, B-cell/TLS organization, tissue-resident immune cells, TAMs, MDSCs, Tregs, eosinophils, and ILC2/type 2 immune programs. Cell-of-origin remains essential because ADAM12 can also function as a T-cell costimulatory molecule influencing Th1 fate in tissue inflammation [55]. This evidence hierarchy supports ADAM12 as a marker and context-specific contributor to immunosuppressive stromal states.

## Major ADAM12-associated signaling pathways: integrated mechanistic synthesis

ADAM12-associated signaling can be organized into recurrent modules that bridge physical, metabolic, and immune pillars. These modules connect tumor progression, stromal remodeling, EMT, immune regulation, and therapeutic resistance across cell types and model systems. Table 1 summarizes the major ADAM12-associated signaling modules, principal cell-of-origin contexts, biological outputs, and evidence bases used throughout this Review.

**Table 1:** Major ADAM12-associated signaling pathways and cell-of-origin contexts in tumor–stromal biology

Pathway module / interpretive context	ADAM12-linked node	Principal cell-of-origin / model context	Biological output	Evidence basis	Representative refs
Protein and isoform framework	ADAM12-L is membrane-associated and contains extracellular prodomain, metalloproteinase, disintegrin-like, cysteine-rich/EGF-like regions, transmembrane segment, and cytoplasmic tail; ADAM12-S lacks the transmembrane and cytoplasmic regions and is generally secreted.	Structural, biochemical, trafficking, isoform-resolved biomarker, and assay-specific contexts.	Defines how ADAM12 measurements differ across mRNA, total protein, cell-surface protein, secreted protein, and assay-specific readouts.	Interpretive protein framework. ADAM12 biology depends on isoform, localization, assay, and cell source.	[30, 33, 38]
TGF-β/SMAD–ADAM12–CAF/myCAF–IFN-I fibroblast-state module	ADAM12 sustains TGF-β-associated myofibroblastic programs and restrains IFN-I-responsive antitumor fibroblast states in selected perturbation systems.	ADAM12-positive CAF/myCAF states; patient-derived fibroblast Perturb-seq; organoid-fibroblast co-culture; fibroblast-specific Adam12 perturbation models.	ECM remodeling, myCAF-state maintenance, reduced IFN-I-responsive fibroblast programs, impaired effector-cell access, and tumor-supportive stromal architecture.	Direct fibroblast perturbation evidence. Establishes ADAM12 as a fibroblast checkpoint in defined CAF/myCAF settings.	[18]
Tumor–stroma TGF-β feedback and tumor-cell ADAM12	Stromal TGF-β1 can induce ADAM12 expression in tumor cells; ADAM12 can participate in EMT-associated tumor-cell programs.	Tumor-cell ADAM12 in breast cancer and EMT-related models; tumor–stroma feedback contexts.	Tumor progression, EMT-associated plasticity, invasion, and tumor–stroma signal reinforcement.	Direct tumor-cell functional evidence plus tumor-stroma feedback support. Distinguishes tumor-cell ADAM12 from CAF-derived ADAM12 biology.	[45, 46]
EGFR/ERK and EMT-associated tumor-cell signaling	ADAM12 contributes to EGFR/ERK-linked EMT, proliferation, migration, and invasion in selected tumor-cell models.	Pituitary adenoma and gastric cancer tumor-cell contexts; tumor-cell knockdown or overexpression systems.	EMT, migration, invasion, proliferation, apoptosis resistance, and adverse clinicopathologic associations.	Direct tumor-cell functional evidence. Supports ADAM12 as a tumor-cell signaling amplifier.	[28, 47]
ADAM12–basigin/CD147–β1 integrin–ECM remodeling	ADAM12 sheds basigin/CD147, and the ADAM12-generated basigin ectodomain engages β1 integrin.	Tumor-cell and matrix-remodeling models with ADAM12-dependent basigin shedding.	Gelatin degradation, fibronectin and COL5A1 expression, collagen-rich stromal remodeling, and desmoplastic matrix reinforcement.	Mechanistic support with in vivo matrix-remodeling evidence. Supports an ADAM12-linked sheddase–integrin–ECM module.	[62, 69]
ADAM12-L–integrin/MMP14–FAK/Src–YAP/TAZ-permissive mechanotransduction	ADAM12-L cooperates with αVβ3 integrin, MMP14, ZO-1, invadopodia-associated structures, focal adhesions, and FAK/Src signaling.	Tumor-cell invasion, EMT, invadopodia, focal adhesion, and matrix-degradation models.	Local matrix degradation, adhesion turnover, invasion, collagen remodeling, apoptosis resistance, FAK/Src activation, and YAP/TAZ-permissive mechanotransduction.	Mechanistic support with tumor-type specificity. Connects ADAM12 to matrix-adhesion signaling and mechanotransduction-associated outputs.	[61, 63, 75, 76]
HIF-1α/NOTCH1–ADAM12–HB-EGF–EGFR/FAK feedback module	Hypoxia activates HIF- and NOTCH-linked signaling; NOTCH1-dependent ADAM12 upregulation increases ADAM12 proteolytic activity, HB-EGF shedding, EGFR activation, FAK phosphorylation, and invadopodia-associated invasion in selected tumor-cell models.	Breast cancer hypoxia model; tumor-cell NOTCH1/HB-EGF/EGFR/FAK feedback contexts; supportive ameloblastoma and adenoid cystic carcinoma expression contexts.	Hypoxia-linked migration, invasion, invadopodia formation, EGFR/FAK activation, and metastatic behavior.	Direct mechanistic support in defined tumor-cell models plus supportive pathway evidence. Defines a HIF/NOTCH–ADAM12–HB-EGF/EGFR feedback loop.	[77, 78]
ADAM12-S–HK1 and ADAM12-L–PI3K/Akt survival or resistance modules	ADAM12-S is associated with HK1 and glycolytic output, whereas ADAM12-L is linked to PI3K/Akt-mediated survival and drug-resistance signaling.	Small-cell lung cancer, breast cancer 5-FU-resistance, lymphoma, choriocarcinoma, and other selected tumor-cell resistance models.	Glycolytic output, proliferation, adhesion, migration, invasion, survival signaling, and therapy tolerance in selected tumor-cell systems.	Direct tumor-cell functional evidence. Links ADAM12 isoforms to glycolytic output, survival signaling, and therapy-tolerant states.	[54, 111, 112, 162]
PDGFRα-positive ADAM12-positive stromal-cell niche	ADAM12 marks slow-cycling PDGFRα-positive mesenchymal stromal cells that support macrophage efferocytosis, pathological angiogenesis, vascular dysfunction, hypoxia/acidosis, and CAF-state maintenance in selected preclinical systems.	PDGFRα-positive ADAM12-positive mesenchymal stromal cells in melanoma, pancreatic cancer, prostate cancer, and pancreatic neuroendocrine cancer models.	Vascular dysfunction, hypoxia, acidosis, macrophage efferocytosis, CAF-state maintenance, effector-cell exclusion, and impaired antitumor immunity.	Direct stromal-cell depletion evidence. Defines an ADAM12-positive stromal compartment with vascular, metabolic, myeloid, and immune consequences.	[44]
ADAM12-positive fibrogenic-cell targeting and stromal normalization	ADAM12-positive fibrogenic cells can be targeted by vaccination or related depletion strategies in preclinical fibrosis-high tumor models.	ADAM12-positive CAF/fibrogenic-cell compartments, especially preclinical PDAC models.	Reduced CAF abundance and desmoplasia, delayed tumor growth, vascular normalization, hypoxia relief, and altered intratumoral CD8⁺ T-cell distribution in selected preclinical systems.	Direct preclinical perturbation evidence. Supports ADAM12-positive fibrogenic-cell targeting as stromal priming.	[90, 164]
Immune-cell ADAM12 and direct immune-relevant perturbation contexts	ADAM12 can function as a T-cell costimulatory molecule in tissue inflammation; tumor-cell Adam12 ablation and fibroblast/stromal-cell ADAM12 perturbation can alter immune composition in selected models.	T-cell ADAM12, tumor-cell Adam12, ADAM12-positive fibroblast checkpoint models, and ADAM12-positive stromal-cell depletion models.	Th1 fate modulation, altered B-cell/T-cell abundance, reduced neutrophil or MDSC enrichment in selected models, improved effector-cell infiltration or T-cell-based immune control in selected preclinical systems.	Direct immune-relevant functional evidence. Cell of origin distinguishes T-cell costimulation, tumor-cell Adam12, fibroblast checkpoint activity, and CAF/MSC-associated immune exclusion.	[18, 44, 55, 129]
Correlative immune-stromal and immunotherapy-related human datasets	ADAM12 expression associates with ECM-high CAF programs, immune/stromal scores, immune checkpoint genes, TMB, MSI, and immunotherapy-related features in pan-cancer or tumor-specific datasets.	Pan-cancer datasets, bladder cancer datasets, LUAD, ccRCC, PDAC, gastric cancer, and other human association studies.	Immunosuppressive or stromal-rich tumor states, checkpoint-gene associations, TME classification, and potential immunotherapy-response stratification.	Human association data. Supports hypothesis generation, patient stratification, and spatial or functional follow-up.	[56, 57, 136, 137]
Contextual immune axes: lymphatics, TDLN priming, DC trafficking, ILC2/type 2 immunity	ADAM12-high stromal states may coexist with tissue ecologies involving lymphatic drainage, antigen transport, DC trafficking, TDLN priming, lactate-sensitive ILC2 impairment, IL-33/ILC2/eosinophil axis, and type 2 immunity.	Tissue-level immune geography and contextual pathway integration.	Antigen transport, immune-cell trafficking, TDLN immune priming, tissue-resident immune programs, type 2 immune regulation, and immunotherapy response context.	Contextual tissue-level framework. Integrates lymphatic, TDLN, ILC2/eosinophil, and type 2 immune biology as modifiers of ADAM12-high stromal states rather than as direct ADAM12 mechanisms.	[24, 119, 124, 126, 141]

Evidence categories: Functional perturbation evidence denotes genetic, pharmacologic, vaccination-based, depletion-based, or cell-state perturbation of ADAM12 or ADAM12-positive compartments with measured biological outputs. Mechanistic support denotes defined substrate, adhesion, protease, or signaling evidence. Correlative human data denote patient-dataset, transcriptomic, spatial, serum, tissue-biomarker, or pan-cancer associations. Contextual tissue-level links denote broader TME processes that position ADAM12-high states within vascular, metabolic, lymphatic, or immune geography. Representative references are grouped by module and should not be read as evidence that each citation supports every output listed in a row. Direct perturbation evidence, mechanistic support, correlative human data, and contextual tissue-level links are separated to avoid causal overinterpretation

*Abbreviations*: *ADAM12* A disintegrin and metalloproteinase 12, *CAF* Cancer-associated fibroblast, *ccRCC* Clear cell renal cell carcinoma, *CD147* basigin, *DC* Dendritic cell, *ECM* Extracellular matrix, *EGFR* Epidermal growth factor receptor, *EMT* Epithelial–mesenchymal transition, *ERK* Extracellular signal-regulated kinase, *FAK* Focal adhesion kinase, *HB-EGF* Heparin-binding EGF-like growth factor, *HIF* Hypoxia-inducible factor, *HK1* Hexokinase 1, *IFN-I* Type I interferon, *ILC2* group 2 innate lymphoid cell, *LUAD* Lung adenocarcinoma, *MDSC* Myeloid-derived suppressor cell, *MMP14* Matrix metallopeptidase 14, *MSI* Microsatellite instability, *MSC* Mesenchymal stromal cell, *PDAC* Pancreatic ductal adenocarcinoma, *SCLC* Small-cell lung cancer, *TAM* Tumor-associated macrophage, *TDLN* Tumor-draining lymph node, *TGF-β* Transforming growth factor-β, *TLS* Tertiary lymphoid structure, *TMB* Tumor mutational burden, *TNBC* Triple-negative breast cancer, *YAP/TAZ* Yes-associated protein/transcriptional coactivator with PDZ-binding motif

### TGF-β/SMAD, CAF/myCAF state control, and IFN-I-responsive fibroblast programs

The most central stromal signaling module links ADAM12 to TGF-β-associated CAF/myCAF biology. In fibroblast and myofibroblast contexts, ADAM12 is associated with TGF-β-high stromal programs, matrix remodeling, and maintenance of pro-tumoral myofibroblastic states. This placement is consistent with broader CAF biology in which TGF-β/SMAD signaling can promote myofibroblast generation, including macrophage-myofibroblast transition in selected stromal models [165]. PDAC single-cell studies further show that CAF compartments include diverse inflammatory, myofibroblastic, and antigen-presenting states, placing ADAM12-positive CAF/myCAF biology within one state-linked module of the CAF landscape [42]. Mechanistically, IL1-driven JAK/STAT signaling and TGF-β signaling can antagonistically shape inflammatory and myofibroblastic CAF states, providing a useful background for interpreting ADAM12 within TGF-β-high myCAF programs [43]. The strongest ADAM12-specific evidence comes from the Li et al. single-cell screening study, which used patient-derived fibroblast perturbation and functional validation to show that ADAM12 ablation can shift fibroblast states away from TGF-β-associated myofibroblastic programs and toward IFN-I-responsive antitumor fibroblast states, with improved T-cell-based immune control and tumor rejection in murine models [18]. This supports ADAM12 as a fibroblast checkpoint and candidate CAF-focused target or reprogramming node. CAF identity, state definition, and therapeutic relevance remain active areas of investigation in cancer immunotherapy and clinical translation [153].

This module also provides a bridge between tumor-cell and stromal-cell ADAM12 biology. TGF-β can induce ADAM12 expression during EMT-associated programs, and breast cancer data support tumor-cell ADAM12 as a tumor-stroma feedback-associated driver of progression [45, 46]. These observations support a bidirectional tumor-stroma feedback model. Tumor-cell ADAM12, CAF-derived ADAM12, and PDGFRα-positive ADAM12-positive mesenchymal stromal-cell programs are therefore considered as distinct but interconnected mechanisms.

### ECM, integrin, MMP14, FAK/Src, and YAP/TAZ-permissive mechanotransduction

A second recurring module connects ADAM12 to ECM remodeling, integrin-associated adhesion, and mechanotransduction. ADAM12-generated basigin/CD147 ectodomain can bind β1 integrin and support gelatin degradation, fibronectin and COL5A1 expression, and collagen-rich ECM remodeling [69]. ADAM12-L can also cooperate with αVβ3 integrin and MMP14 at the cell surface, supporting localized matrix degradation and adhesion-linked survival or invasion phenotypes [63]. These findings provide mechanistic support for ADAM12 as an organizer of selected ECM-integrin-protease modules.

Downstream of this matrix-adhesion interface, FAK/Src signaling provides a major intracellular route. ADAM12-L has been linked to focal adhesion turnover and FAK activation in esophageal cancer models, with FAK-JNK-c-Jun signaling feeding back to sustain ADAM12-L transcription [75]. ADAM12-mediated ephrin-A1 cleavage illustrates how proteolytic output can extend beyond matrix remodeling into vascular or permeability-related phenotypes [166]. ADAM12-linked STAT3-dependent tumor angiogenesis provides a separate vascular-output example [167]. Together, these mechanisms connect ECM remodeling, substrate cleavage, and adhesion dynamics to mechanotransduction and vascular signaling in selected models.

### Hypoxia, NOTCH, HB-EGF/EGFR, EMT, and tumor-cell survival signaling

A third module links ADAM12 to hypoxia-responsive and growth-factor signaling in selected tumor-cell models. Under hypoxia, HIF-dependent ADAM12 induction can promote HB-EGF shedding, EGFR activation, FAK phosphorylation, migration, invasion, and metastatic behavior in breast cancer models [77]. NOTCH signaling adds a distinct regulatory layer. In hypoxic tumor-cell contexts, NOTCH activation increases ADAM12 expression and proteolytic activity; ADAM12 then sheds HB-EGF, which activates EGFR and downstream FAK signaling to support invadopodia formation and invasive behavior [78]. This creates a Notch–ADAM12–HB-EGF/EGFR feedback loop that couples cell-contact-dependent signaling to paracrine growth-factor activation.

Other tumor-cell modules include EGFR/ERK, PI3K/Akt, HK1, NF-κB/MGMT, STAT3/VEGFA, EMT, invasion, and therapy-resistance pathways. ADAM12 silencing impaired tumorigenic and EMT-associated behavior through EGFR/ERK signaling in pituitary adenoma models [47]. ADAM12-S has been linked to HK1 expression and glycolytic output in small-cell lung cancer models [111]. ADAM12-L has been linked to PI3K/Akt-associated acquired 5-fluorouracil resistance in breast cancer models [162]. Glioblastoma resistance is a separate TNF-α/NF-κB/MGMT-associated temozolomide-resistance context [148]. These findings define tumor-type-specific signaling modules rather than a pan-cancer ADAM12 resistance pathway.

### Immune, inflammatory, and correlative pan-cancer signaling contexts

ADAM12-associated immune signaling should be separated into direct perturbation evidence, immune-cell ADAM12 biology, and broader ADAM-family context. T-cell ADAM12 costimulation represents a distinct immune-cell setting [55]. Tumor-cell Adam12 ablation can alter immune composition in selected models [129]. ADAM-family sheddases can also regulate tumor immunity through non-ADAM12 mechanisms; targeting the ADAM17-CD122 axis enhances CD8 + T-cell effector differentiation and antitumor immunity in solid-tumor models [168]. This framework supports discussion of ADAM12 within immune regulation only when isoform, family member, substrate, and cell of origin are specified.

Human datasets provide a complementary evidence layer. Pan-cancer analysis is best used for hypothesis generation about immune-stromal ecology rather than causal inference [56]. Bladder cancer work links ADAM12 to an ECM-high CAF program and immune-excluded microenvironment [57]. These datasets support the relevance of ADAM12 to immune-stromal tumor ecology and provide hypotheses for spatial localization, perturbation experiments, and biomarker-guided patient stratification.

Broader immune axes, including lymphatic drainage, tumor-draining lymph node priming, dendritic-cell trafficking, and B-cell/TLS organization, are integrated here as tissue-level immune context [124]. Lactate-sensitive ILC2 impairment adds a metabolic immune-context axis [119]. Type 2 immunity provides a broader tissue-level immune framework [126]. These processes help explain why ADAM12-high stromal states should be interpreted within a broader tissue-level immune geography, while direct ADAM12 dependence must be tested separately.

In summary, major ADAM12-associated signaling pathways can be organized into four groups: a TGF-β/CAF/myCAF module with IFN-I-responsive fibroblast-state restraint; an ECM-integrin-MMP14-FAK/Src mechanotransduction module; a hypoxia/NOTCH/HB-EGF/EGFR and tumor-cell survival module; and an immune-stromal context module that combines perturbation studies with human datasets. This synthesis improves mechanistic readability and emphasizes that ADAM12 functions as an isoform-aware, cell-of-origin-dependent marker, amplifier, or perturbable node within tumor-stromal programs.

## Clinical implications: biomarkers and resistance readouts

The translational value of an ADAM12-centered stromal-checkpoint framework depends on whether ADAM12 activity or abundance can be measured, stratified by isoform and cell of origin, and linked to clinically meaningful barrier biology. Clinical interpretation rests on how ADAM12 functions as a prognostic or stromal-state readout, how tissue and biofluid assays differ, and how ADAM12-associated resistance signals can support biomarker-guided combination hypotheses. Most clinical datasets remain associative, positioning ADAM12 as a context-resolved readout and candidate sensitization node for high-barrier tumor ecosystems (Table 2).

**Table 2:** Biomarker and therapeutic implications of ADAM12-associated barrier biology

Translational module	Candidate application / strategy	Matching requirements	Evidence status and limitation	Refs.
Tissue, serum, plasma, urine, or transcript-based ADAM12 biomarkers	Use ADAM12 as a stromal-state or aggressive-disease biomarker and as a potential longitudinal monitoring signal.	Define sample type, protein versus mRNA readout, isoform coverage, assay species, tissue localization, CAF-state markers, ECM/fibrosis signatures, and confounding inflammatory or fibrotic disease states.	Clinical association and investigational monitoring evidence. Best used as part of stromal-state and longitudinal biomarker panels.	[26, 89, 92, 161]
ADAM12-positive fibrogenic-cell vaccination or tag-based depletion	Fibrolytic vaccination or related strategies to target ADAM12-positive fibrogenic compartments in fibrosis-high tumors.	Requires ADAM12-positive CAF/fibrogenic-cell enrichment, tumor-restricted or disease-restricted expression, and safety assessment for normal tissue remodeling.	Preclinical proof-of-principle evidence. In PDAC, published effects include delayed tumor growth, reduced CAFs/desmoplasia, vascular normalization, reduced hypoxia, and T-cell redistribution.	[90, 164]
Fibroblast-checkpoint reprogramming	Perturb ADAM12 to shift TGF-β-high myCAF states toward IFN-I-responsive, progenitor-like, antitumor fibroblast states; consider ICB sensitization where this state transition is present.	ADAM12-positive myCAF/TGF-β-high fibroblast states; ProgB/ProgC or equivalent IFN-I/TGF-β readouts; spatial T-cell exclusion; PD-L1 or other established ICB biomarkers.	Direct preclinical fibroblast perturbation evidence. Clinical translation, antibody development, treatment window, combination sequence, and detailed stromal-immune mechanisms remain unresolved.	[18]
PDGFRα⁺ADAM12⁺ stromal-cell normalization	Target or deplete PDGFRα⁺ADAM12⁺ MSC-like stromal cells to normalize vasculature, hypoxia, acidosis, CAF state, and myeloid immunosuppression.	Demonstrate PDGFRα⁺ADAM12⁺ MSC enrichment, macrophage efferocytosis or Gas6/Lgals3/Csf1-like programs, vascular dysfunction, hypoxia/acidosis, and effector-cell exclusion.	Direct genetic-depletion evidence in selected preclinical models. Represents context-specific stromal normalization.	[44]
Surface, sheddase, and substrate-level targeting	Block defined ADAM12-linked extracellular interfaces or use ADAM12-rich surfaces for conditional delivery. Candidate substrate modules include HB-EGF/EGFR, basigin–β1 integrin, ephrin-A1, and ADAM12-S-dependent secretome remodeling.	Confirm accessible ADAM12 species, isoform, cell of origin, substrate dependence, extracellular localization, and pathway activation in the target tumor.	Mechanistic or preclinical rationale. Selective inhibition, payload delivery, substrate redundancy, and normal-tissue toxicity require validation.	[62, 69, 77, 78, 166]
Downstream mechanochemical cotargeting	Combine ADAM12-informed stromal profiling with FAK/Src, YAP/TAZ, MMP14/integrin, MEK/ERK, Notch, or EGFR pathway cotargeting.	Requires pathway evidence such as activated adhesion/stiffness signaling, HIF/Notch activity, EGFR signaling, MEK/ERK activity, collagen remodeling, or MMP14/integrin engagement.	Combination rationale. Apply when ADAM12-high tumors show active downstream matrix-adhesion, stiffness, or growth-factor signaling.	[63, 75, 76]
Tumor-cell resistance sensitization	Pair ADAM12-linked tumor-cell pathway modulation with 5-FU, gemcitabine, EGFR-TKIs, temozolomide, or radiotherapy in defined tumor settings.	Confirm ADAM12 isoform and tumor-cell origin; verify EGFR/ERK, PI3K/Akt, NF-κB/MGMT, FOXC2, STAT3, EMT, or radiation-induced ADAM12 pathway activity.	Direct evidence in selected tumor-cell models. Supports tumor-type-specific resistance-sensitization hypotheses.	[148, 162, 165, 169–172]
Regulatory-state reset	Use RNA, miRNA, chromatin, epigenetic, or EV-related strategies to suppress ADAM12 or ADAM12-containing regulatory programs when direct regulation and delivery feasibility are demonstrated.	Demonstrate direct miRNA or chromatin regulation, isoform affected, delivery cell type, biodistribution, and functional endpoint. Candidate axes include miR-29/miR-200/miR-30, NSD3/JMJD2A, HDAC-sensitive secretome changes, and EV-based delivery.	Mostly preclinical or conceptual. Requires rigorous delivery, biodistribution, and vesicle-characterization standards.	[66, 151, 155, 173–175]
Composite immune-response monitoring beyond ADAM12 alone	Use ADAM12 as one component of a biomarker panel for stromal priming plus ICB or other immune combinations.	Assess spatial immune exclusion, CAF/myCAF and myeloid organization, B cells/TLS, lymphatic drainage, TDLN priming, PD-L1, MSI, TMB, CD8⁺ T-cell function, and context-dependent ILC2/eosinophil or type 2 immune programs.	Composite biomarker strategy. ADAM12 should be integrated with spatial immune, lymphatic/TDLN, TLS, type 2 immunity, and established ICB-response readouts.	[22, 134, 138]

Representative references in this table are grouped by therapeutic module and support the evidence category, rationale, and validation status summarized in each row. No row should be read as evidence that ADAM12 directly regulates every listed biomarker, immune feature, or therapeutic output

*Abbreviations*: *5-FU* 5-fluorouracil, *ADAM12* A disintegrin and metalloproteinase 12, *CAF* Cancer-associated fibroblast, *ECM* Extracellular matrix, *EGFR* Epidermal growth factor receptor, *EGFR-TKI* Epidermal growth factor receptor tyrosine kinase inhibitor, *EMT* Epithelial-mesenchymal transition, *EV* Extracellular vesicle, *FAK* Focal adhesion kinase, *HB-EGF* Heparin-binding EGF-like growth factor, *ICB* Immune checkpoint blockade, *IFN-I* Type I interferon, *ILC2* group 2 innate lymphoid cell, *MGMT* O⁶-methylguanine-DNA methyltransferase, *MMP14* Matrix metallopeptidase 14, *MSC* Mesenchymal stromal cell, *MSI* Microsatellite instability, *PDAC* Pancreatic ductal adenocarcinoma, *TDLN* Tumor-draining lymph node, *TLS* Tertiary lymphoid structure, *TMB* Tumor mutational burden, *TMZ* Temozolomide, *YAP/TAZ* Yes-associated protein/transcriptional coactivator with PDZ-binding motif

### Pan-cancer prognostic associations and stromal-state interpretation

Across multiple tumor types, high ADAM12 expression is associated with stromal remodeling, mesenchymal differentiation, invasive behavior, and adverse clinical outcome, but the biological meaning of this association varies by tumor type, isoform, and cell of origin. PDAC tissue ADAM12 expression has been linked to shorter disease-free and overall survival after surgical resection and perioperative chemotherapy [176]. Circulating ADAM12 behaves as a blood-borne proxy for stromal activation and treatment benefit in clinical cohorts [89]. These data support ADAM12 as a clinically informative stromal-state readout.

Across gastrointestinal, thoracic, ovarian, breast, and gastric cancer datasets, ADAM12 captures both stromal and tumor-cell biology. Colorectal and colon cancer datasets place ADAM12 in prognostic programs associated with advanced stage, nodal involvement, relapse, and shorter survival [177, 178]. Serum-based measurements of ADAM12 in metastatic colorectal cancer also associate stromal activation with poor prognosis, supporting a blood-based stromal readout in this disease context [161]. Functional colorectal cancer studies link ADAM12 to EMT, invasion, peritoneal metastasis, and PI3K/Akt-associated signaling, supporting tumor-cell mechanistic roles in models [179]. Additional colorectal cancer data connect ADAM12 overexpression to Wnt/β-catenin-associated EMT and tumor growth [180]. Gastric cancer studies link ADAM12 overexpression to tumor progression, metastasis, immune infiltration, Akt/GSK-3β signaling, and worse outcome, combining clinical association with tumor-cell functional support [28, 181]. Secretome proteomics and biochemical analyses further showed that ADAM12-S can regulate gastric cancer cell migration through CD146-dependent mechanisms, reinforcing isoform-specific tumor-cell interpretation in gastric cancer [182]. Matrine-related ceRNA network analysis also identified ADAM12 and PDGFRB as gastric cancer biomarkers linked to ECM protease activity and immune infiltration [183]. Hepatocellular carcinoma data further connect TSPAN8-driven ADAM12m expression to invasion, metastasis, and poor survival [51]. Thoracic cancer datasets, including LUAD and SCLC, associate ADAM12-containing programs with poor survival, invasive behavior, immune-related pathway enrichment, and HK1-linked tumor-cell phenotypes in models [111, 136]. Ovarian cancer evidence aligns ADAM12 with mesenchymal and metastatic programs, with isoform-aware interpretation remaining important [184]. ADAM12 isoforms can confer estrogen-independent growth in breast cancer cells [49]. ADAM12 has also been associated with a breast tumor-initiating cell phenotype [149].

These clinical observations reflect multiple ADAM12-containing biological programs. In some tumors, ADAM12 primarily reports CAF-rich or ECM-high stromal states; in others, it reflects tumor-cell EMT, growth-factor shedding, glycolytic adaptation, or therapy-induced survival signaling. Recent bladder cancer and pan-cancer analyses associate ADAM12 with ECM-high CAF programs, immune and stromal scores, immune checkpoint genes, TMB, MSI, and immunotherapy-related features [56, 57]. In renal cancer, obesity-associated peritumoral adipose tissue can induce ADAM12 and CYP1B1 in adipose-derived stromal contexts and promote tumor-cell invasiveness, illustrating that ADAM12-high stromal ecology may also involve adipose-TME interactions outside classical CAF definitions [185]. HLA-G neo-expression studies in clear cell renal carcinoma further indicate that immune-checkpoint-associated tumor-cell programs can modify ADAM12-containing genetic networks [147].

Clinical evidence supports a stratified conclusion. ADAM12 can mark high-risk, high-barrier tumor ecosystems, and selected models support mechanistic roles in invasion, stromal remodeling, metabolic adaptation, or immune suppression. Clinical use will require integration with histology, CAF-state markers, spatial immune profiling, isoform information, treatment context, and longitudinal response data.

### Biofluid applications: liquid biopsy, assay specificity, and longitudinal surveillance

The clinical appeal of ADAM12 is enhanced by the fact that it can be measured outside tissue sections, although biofluid use remains investigational and context-dependent. Depending on the assay and sample type, ADAM12-related signals may reflect secreted ADAM12-S, shed extracellular species, circulating protein, mRNA, or extracellular-compartment-associated material; therefore, assay format and isoform coverage should be reported explicitly [30, 41]. This is especially relevant for stromal checkpoint biology, because tissue sampling may undersample spatial heterogeneity or fail to capture dynamic treatment-associated changes.

Serum and plasma studies provide the most developed, but still heterogeneous, translational evidence. In PDAC, circulating ADAM12 functions as a blood-borne proxy for stromal activation and carries prognostic and treatment-response information under chemotherapy [89]. In hepatobiliary malignancies, elevated serum ADAM12 has been evaluated as an adjunct marker to distinguish HCC from cholangiocarcinoma, supporting diagnostic feasibility in a specific differential setting [186]. In esophageal squamous cell carcinoma, ADAM12 was among serum candidates elevated in patients, although the final high-performing diagnostic model relied on a multi-marker strategy [93]. In metastatic colorectal cancer, serum-based ADAM12 stromal-activation measurements have been associated with poor prognosis [161]. In cervical cancer, tissue and serum ADAM12 expression have been associated with invasion, TNM stage, lymph-node metastasis, differentiation, and poorer survival, supporting additional tumor-type-specific biomarker feasibility [154]. Together, these data show that ADAM12 can be measured in blood and may be clinically informative, with tumor specificity, cutoff selection, assay platform, and non-cancer inflammatory or fibrotic confounders requiring validation.

Dynamic tracking may be one of the most useful features of circulating ADAM12, but this application remains less mature than simple baseline association. Serial ADAM12 measurements should therefore be interpreted in tumor-type-specific cohorts rather than generalized across cancers. In PDAC, serial ADAM12 measurements during chemotherapy provide a stronger example of treatment-linked monitoring because decreasing or persistently undetectable levels were associated with better outcome. In colorectal cancer, multi-omics analyses of EMT-linked surface proteins have also identified ADAM12 among candidate genes associated with EMT and lipid-metabolic programs, reinforcing the need to connect biomarker interpretation to pathway context [187]. This characteristic is particularly important for a stromal biomarker, because the clinically relevant question is often not whether a tumor ever had stromal activation, but whether such activation is intensifying, persisting, or being therapeutically modulated during treatment. Future use should therefore pair serial ADAM12 measurements with imaging, CAF-state markers, perfusion or hypoxia readouts, EMT/metabolic pathway readouts, and treatment-response endpoints.

Overall, current evidence supports ADAM12 as a feasible investigational non-invasive biomarker whose greatest potential may lie in longitudinal monitoring and treatment stratification of stromal-high tumors.

### Mechanisms and readouts of therapy resistance

The clinical importance of ADAM12 does not end with prognosis or surveillance. In defined models, ADAM12 has been functionally linked to therapy resistance through isoform-specific growth-factor, PI3K/Akt, EGFR/ERK, NF-κB, STAT3, or EMT-associated signaling. In other settings, ADAM12 serves as a tissue or circulating marker of CAF-rich, hypoxic, fibrotic, acidic, immune-excluded, or otherwise immunosuppressive barrier states that are themselves associated with poor treatment response. Thus, ADAM12 may function as both a resistance amplifier in defined models and a resistance readout in selected clinical or stromal contexts.

Direct tumor-cell resistance evidence is strongest in defined models. In breast cancer, ADAM12-L can confer acquired 5-fluorouracil resistance through PI3K/Akt-associated signaling [162]. In bladder cancer, ADAM12 has been linked to gemcitabine resistance through EGFR pathway activation and EMT induction [169]. In glioblastoma, ADAM12 promotes temozolomide resistance through TNF-α shedding, NF-κB activation, MGMT regulation, and apoptosis resistance, and ADAM12 inhibition sensitizes tumors in preclinical systems [148]. In LUAD, ADAM12 has been associated with EGFR-TKI resistance and PI3K/Akt/mTOR or RAS pathway activity [170]. In rectal cancer, ADAM12 expression is upregulated after radiation and has been proposed as a prognostic factor after radiotherapy, with preclinical data suggesting links to tumor vascularity and treatment response [165]. These examples illustrate a general principle: treatment itself can intensify mesenchymal, adhesive, vascular, or barrier-like states if compensatory signaling is left unchecked. In ADAM12-high disease, this provides a rationale for pairing standard therapies with agents that intercept the adaptive pathway demonstrated in that tumor.

Stromal-mediated resistance forms a parallel clinical category. In esophageal adenocarcinoma, IL-6-producing CAFs drive EMT and treatment resistance, while serum ADAM12 functions as a stromal marker associated with poor response to neoadjuvant therapy [91]. In PDAC and other fibrosis-rich settings, ADAM12-positive CAF or MSC compartments are associated with desmoplasia, poor perfusion, hypoxia, acidosis, and immune exclusion in preclinical or clinical contexts. These data support ADAM12 as a stromal-resistance readout rather than a stand-alone resistance mechanism.

Taken together, ADAM12 occupies a clinically important position at the boundary between biomarker and mechanism. ADAM12-L has direct evidence in selected 5-FU resistance models; ADAM12-associated EGFR, PI3K/Akt, NF-κB, STAT3, and EMT programs support selected chemo-, targeted-, and radioresponse mechanisms; serum ADAM12 can report stromal activation and treatment outcome in selected gastrointestinal cohorts; and ADAM12-positive CAF or MSC compartments can sustain vascularly abnormal, hypoxic, acidic, and immune-excluded or otherwise immunosuppressive niches in preclinical systems. ADAM12 is therefore relevant for precision oncology as a context-resolved readout and potential sensitization node to be interpreted together with isoform status, cell of origin, CAF-state markers, spatial immune profiling, perfusion or hypoxia readouts, pathway activity, and longitudinal treatment-response data.

## Therapeutic strategies: stromal priming and combination therapy

**Fig. 3:**
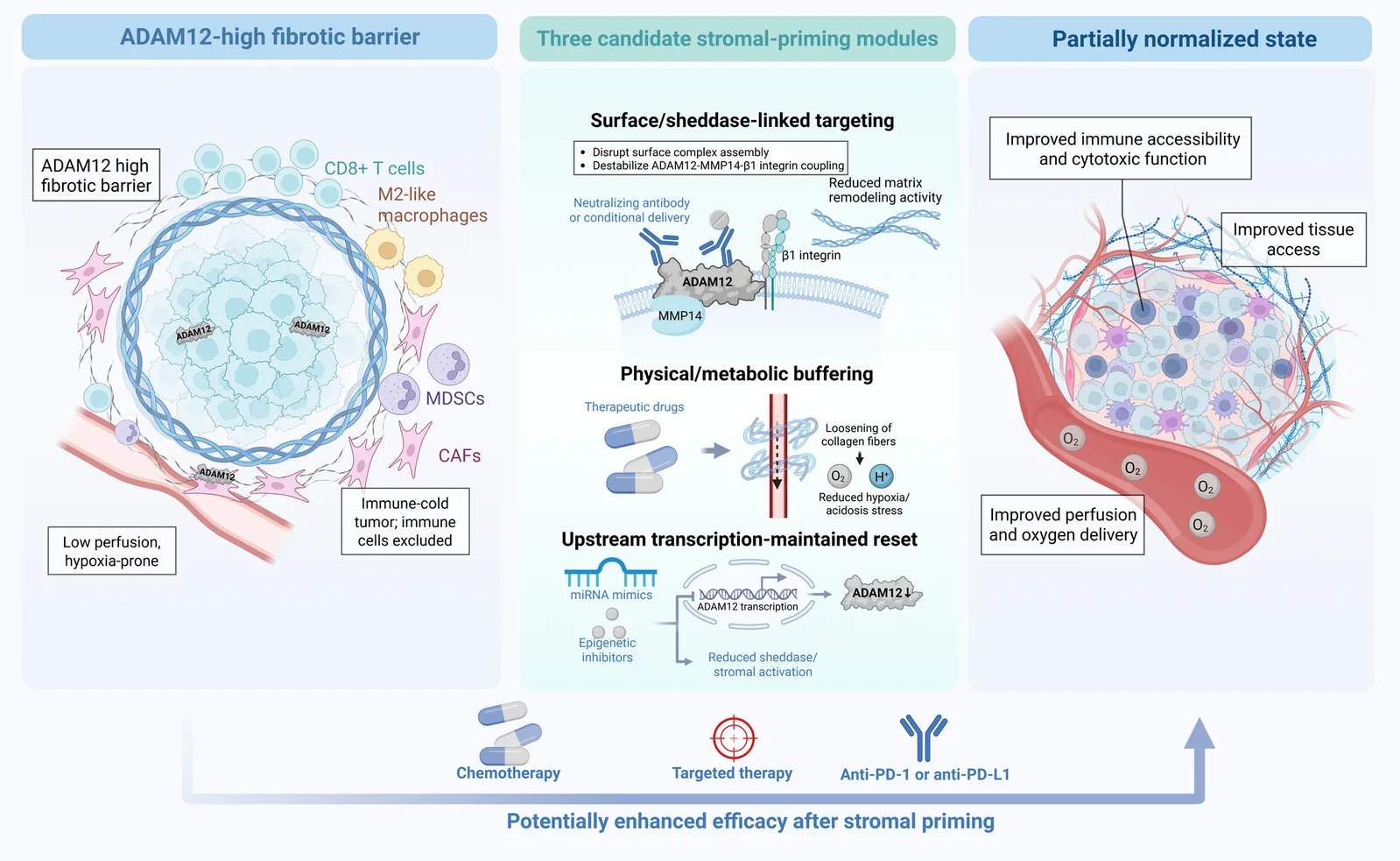
Candidate stromal-priming strategies for ADAM12-associated high-barrier tumor states. ADAM12-associated high-barrier tumor states provide a framework for candidate stromal priming. The left panel depicts an ADAM12-high fibrotic barrier with fibrotic ECM, low perfusion, hypoxia-prone vasculature, immune-cold architecture, and limited immune access. The center panel summarizes three candidate stromal-priming modules shown in the figure. The first is surface/sheddase-directed targeting, including candidate antibody or conditional-delivery approaches and disruption of ADAM12-MMP14-β1 integrin coupling. The second is physical/metabolic buffering, including loosening of collagen fibers and reduced hypoxia/acidosis stress. The third is regulatory-state reset, including miRNA mimics, epigenetic inhibitors, ADAM12 transcriptional regulation, and reduced sheddase/stromal activation. The right panel depicts a partially normalized state with improved immune accessibility and cytotoxic function, improved tissue access, and improved perfusion and oxygen delivery. The lower axis indicates that stromal priming may potentially enhance chemotherapy, targeted therapy, and anti-PD-1/anti-PD-L1 immunotherapy combinations in selected contexts. Figure created with BioRender.com. Abbreviations: ADAM12, A disintegrin and metalloproteinase 12; CAFs, Cancer-associated fibroblasts; ECM, Extracellular matrix; MDSCs, Myeloid-derived suppressor cells; miRNA, microRNA; MMP14, matrix metallopeptidase 14; PD-1, Programmed cell death protein 1; PD-L1, Programmed death-ligand 1

ADAM12-high barrier states are sustained by intertwined physical, metabolic, vascular, and immune programs, so therapeutic strategies should be framed as candidate stromal priming (Fig. 3) rather than uniform ADAM12 inhibition. Preclinical evidence supports several intervention concepts, including ADAM12-positive fibrogenic-cell vaccination, ADAM12-positive stromal-cell depletion, fibroblast-checkpoint reprogramming, and tumor-cell ADAM12 targeting in defined resistance models [90]. ADAM12-L-associated 5-FU resistance is supported in breast cancer models [162]. Glioblastoma temozolomide resistance represents a separate TNF-α/NF-κB/MGMT-associated context [148]. Therapeutic translation will depend on isoform, cell of origin, tumor type, extracellular accessibility, pathway activity, spatial immune exclusion, and established immunotherapy biomarkers.

### Surface/sheddase targeting and conditional delivery strategies

The most direct, but still clinically immature, strategy is to target ADAM12-associated extracellular biology. As a surface-associated or secreted protease enriched in fibrogenic, invasive, and selected tumor-cell programs, ADAM12 offers two conceptual opportunities: interference with defined proteolytic or adhesion-linked functions, and selective delivery of payloads to ADAM12-rich stromal, vascular, or tumor-cell compartments. ADAM12-mediated ephrin-A1 cleavage provides one example of a defined substrate-linked mechanism with potential relevance to metastasis-associated vascular permeability and biomarker interpretation [166]. ADAM12 has also been reported to regulate tumor angiogenesis through STAT3 signaling, adding a vascular-output context for ADAM12-linked targeting [167]. These observations support substrate- and context-defined targeting hypotheses that remain preclinical.

Several considerations make indiscriminate ADAM12 inhibition risky. ADAM and MMP family members share related metalloproteinase features, substrate overlap, and roles in normal tissue remodeling, which complicates selective catalytic targeting [43, 184]. Experience with better-studied family members reinforces this caution: ADAM10 cleaves many substrates relevant to cancer and immunity, including Notch-related and immune-recognition molecules, while ADAM17 regulates numerous inflammatory, cytokine, receptor, and growth-factor shedding events [36, 188]. ADAM12 itself may also appear in inflammatory or fibrotic non-cancer states, so tumor-restricted enrichment, accessible extracellular epitopes, isoform distribution, and cell-of-origin specificity must be defined before therapeutic targeting is justified [189].

A measured strategy is to treat ADAM12 as a phenotype anchor for conditional targeting. Surfaceome and tumor-vasculature studies support the broader concept that disease-enriched extracellular or vascular markers can be used for imaging, antibody-guided targeting, ligand-guided delivery, radionuclide delivery, cytokine delivery, or payload delivery approaches [190–192]. For ADAM12, this remains a conceptual and preclinical strategy that requires confirmation that the relevant ADAM12 species is accessible, disease-enriched, biologically linked to the barrier state, and safe to target.

Taxane-based treatment is best discussed as a biomarker-linked combination context. In PDAC and esophagogastric adenocarcinoma, circulating or tissue ADAM12 has been associated with stromal activation, outcome, and treatment benefit in nab-paclitaxel-containing or taxane-related clinical contexts [89, 92, 176]. These observations suggest that ADAM12 may help identify tumors in which stromal barrier biology modifies response to taxane-containing regimens.

Overall, surface-directed ADAM12 strategies should be conceptualized broadly but cautiously. Potential approaches include catalytic or allosteric inhibition, blockade of defined shedding networks, substrate-specific interference, stromal- or vascular-directed payload delivery, and pathway-matched combinations. The major translational challenge is selectivity: clinical value will depend on targeting tumor-restricted or disease-enriched ADAM12 biology while avoiding broad chronic protease inhibition in normal tissue homeostasis.

### Downstream mechanochemical and metabolic buffering

Even successful modulation of ADAM12-associated surface or sheddase biology may not immediately erase the downstream consequences of long-standing barrier formation. Once ECM remodeling, stiffness sensing, hypoperfusion, hypoxia, acidosis, and metabolic adaptation are established, tumors can remain protected by mechanochemical and metabolic buffering. This is consistent with broader evidence that ECM remodeling can influence treatment outcome and that cancer therapies themselves can induce ECM changes that contribute to resistance and tumor progression [193]. Downstream vulnerabilities are therefore natural combination partners for ADAM12-informed therapy.

Canonical mechanotransduction provides one rational cotargeting route. FAK is a central mediator of matrix-derived survival and invasion signals [194]. ADAM12-L-FAK feedback provides a tumor-cell example of this linkage [75]. FAK inhibition should therefore be viewed as a downstream pathway-matched option when ADAM12-high tumors show activated matrix-adhesion signaling.

YAP/TAZ-, MEK/ERK-, EGFR-, Notch-, PI3K/Akt-, and hypoxia-associated pathways may provide additional cotargeting opportunities when supported by tumor-specific evidence. For example, ADAM12-containing stromal or ECM signatures may help nominate MEK/ERK or stiffness-associated vulnerabilities, whereas HIF/NOTCH–ADAM12–HB-EGF/EGFR activity may support growth-factor cotargeting when this pathway is demonstrated in a tumor-specific context. These examples define pathway-matched opportunities.

Metabolic buffering provides another combination layer. HK1, PI3K/Akt, hypoxia, lactate, acidosis, and nutrient-stress pathways may coexist with ADAM12-high states, but intervention should be guided by measured metabolic readouts, perfusion or hypoxia status, and pathway activity. This logic identifies tumors in which ADAM12-associated stromal or tumor-cell programs overlap with actionable metabolic dependencies.

These data support rational combination hypotheses in which ADAM12-informed barrier profiling is paired with inhibitors of stiffness signaling, hypoxia adaptation, EMT, vascular dysfunction, growth-factor signaling, or metabolic compensation when the corresponding pathway is active. This combination logic treats ADAM12 as an entry point into a broader resistance network.

### Regulatory-state reset: transcriptional, rna, and epigenetic reprogramming

Durable weakening of ADAM12-high barrier states may require more than transient pathway inhibition. Regulatory-state reset is a future-oriented combination concept aimed at suppressing the transcriptional, post-transcriptional, epigenetic, and microenvironmental reinforcement that maintains EMT, fibroinflammatory signaling, CAF-state plasticity, hypoxia, and therapy-induced survival programs [81, 195, 196]. The therapeutic goal is to prevent re-emergence of the barrier state after stromal priming, not to erase all fibroblasts or chronically suppress physiological tissue remodeling.

At the transcriptional level, the most actionable ADAM12-specific examples remain model-specific. In head and neck squamous cell carcinoma, FOXC2 binds the ADAM12 promoter, FOXC2 silencing lowers ADAM12 expression and increases radiosensitivity, and ADAM12 overexpression can partially reverse the effect of FOXC2 knockdown [197]. This supports transcription-factor-level intervention as a way to reverse an ADAM12-dependent radioresponse module in a defined tumor-cell context. In meningioma models, FOXC2-mediated ADAM12 regulation has also been linked to JAK1/STAT3/VEGFA signaling, proliferation, migration, and invasion, suggesting another tumor-cell-specific transcriptional route into pro-angiogenic or invasive signaling [171]. More broadly, this logic is consistent with the view that phenotypic plasticity and nonmutational reprogramming are central hallmarks of therapy-resistant cancer, and that reversible chromatin states can help tumors survive stromal, metabolic, and therapeutic stress [195, 196].

Post-transcriptional regulation offers an additional therapeutic layer. In oral squamous cell carcinoma cells, miR-29a-3p targets ADAM12, and miR-29a-3p restoration enhances radiosensitivity by suppressing viability, proliferation, and migration while increasing apoptosis [173]. In breast cancer, miR-29 and miR-200 family members directly regulate ADAM12-L, supporting isoform-aware RNA regulation of ADAM12-linked mesenchymal programs [66]. In triple-negative breast cancer, miR-30b has also been reported to suppress ADAM12, adding another RNA-level example of ADAM12 regulation in a breast-cancer context [155]. This is mechanistically attractive because miRNA and lncRNA circuits can intersect with ECM production, fibrosis, mesenchymal state control, and tumor-cell plasticity, making them plausible regulators of ADAM12-containing barrier programs. Other lncRNAs such as DSCAM-AS1 and HNF1A-AS1 belong to broader oncogenic or EMT-associated regulatory networks [160, 198]. The value of RNA-level strategies is strongest when the ADAM12 target relationship, isoform affected, delivery cell type, and phenotypic endpoint are experimentally defined.

Chromatin- and RNA-directed therapies are relevant to ADAM12-centered biology, but ADAM12-specific examples should be separated from broader regulatory-state concepts. In pancreatic cancer models, NSD3 silencing or knockout reduces H3K36 dimethylation, suppresses malignant phenotypes, and decreases expression of NSD3-dependent genes including Adam12 and Notch3 [174]. JMJD2A can transactivate ADAM12 in selected cancer-cell contexts [151]. Histone deacetylase inhibition in glioblastoma cells can alter ECM-associated secretome proteins, including ADAM12 [175]. More broadly, m6A methylation and epigenetic therapies regulate therapy resistance, antigen presentation, immune-cell function, fibroblast activation, EMT, and immune exclusion. These observations support regulatory-state reprogramming as a candidate strategy only when the relevant circuitry is demonstrated.

### Biomarker-to-therapy matching and dynamic response monitoring

ADAM12 is best used as a treatment-matching and monitoring node rather than a stand-alone therapeutic target. High tissue or circulating ADAM12 may identify tumors with stromal buffering, but intervention choice should depend on the accompanying biology. Fibroblast-checkpoint reprogramming is most relevant when ADAM12-positive TGF-β-high myCAF states, IFN-I-responsive fibroblast-state suppression, and spatial immune exclusion are demonstrated. Surface or sheddase-directed strategies require extracellular accessibility and defined substrates; FAK, YAP/TAZ, MEK, Notch, EGFR, PI3K/Akt, or metabolic cotargeting requires evidence that the corresponding downstream pathway is active. Regulatory-state reset is most appropriate when ADAM12-high states are maintained by durable transcriptional, RNA, or chromatin programs.

Immunotherapy combinations require especially careful matching. Candidate combinations should be guided by spatial immune exclusion, CAF/myeloid organization, lymphatic or TDLN-related features, B-cell/TLS organization, type 2 immune context, tissue-resident immune-cell states, and established biomarkers such as PD-L1, TMB, MSI, antigen-presentation status, and T-cell functional state. IL-10-mediated metabolic reprogramming can enhance effector function in terminally exhausted CD8 + TILs [25]. Lactate transport can support stem-like CD8 + T-cell properties or enforce exhausted T-cell dysfunction depending on cellular context [121]. TDLN PD-1/PD-L1 signaling and TLS-associated B-cell organization should be evaluated as separate immune-geography variables [134, 138]. Treg positioning and GDF-15-associated exclusion provide additional non-ADAM12 resistance axes [199, 200]. ADAM12-associated stromal barriers should therefore be analyzed together with metabolic immune fitness, T-cell functional state, TDLN/TLS organization, regulatory-cell geography, and additional TME-derived exclusion signals.

Dynamic models will be needed to move this framework from association to intervention. Organoid, co-culture, spatial explant, and ex vivo tumor-immune models can test whether ADAM12-high barrier features are functionally modulated by protease-directed therapy, stromal normalization, vaccine-based approaches, pathway cotargeting, or checkpoint-sensitizing combinations [201]. These models should also test whether intended barrier biology actually changes after treatment, including ECM density, perfusion, hypoxia, acidosis, myeloid organization, effector-cell positioning, lymphatic drainage, TDLN priming, and type 2 immune programs. Spatial technologies are particularly important for this task because they can connect cell-state abundance with physical location, cell-cell interaction, and therapy-response niches within intact tumor tissue [132].

The practical value of ADAM12-informed therapy will depend on linking each ADAM12 readout to a specific combination strategy and monitoring the corresponding barrier readouts longitudinally. ADAM12 thereby functions as a treatment-matching and monitoring node for high-barrier tumors.

## Conclusion and future outlook

A stromal-checkpoint framework positions the TME as an active resistance ecology. Across the evidence synthesized in this Review, ADAM12 emerges as a context-dependent marker and amplifier of high-barrier tumor-stromal states, with functional regulatory roles in selected models. Its strongest mechanistic support lies in collagen-rich matrix remodeling, integrin/MMP14-centered adhesion biology, fibroblast or mesenchymal stromal-cell programs, hypoxia-linked HB-EGF/EGFR signaling, and immune-state remodeling after direct perturbation. The central conclusion is that ADAM12 biology is most informative when interpreted through isoform, cell-of-origin, and tissue context.

Several take-home messages follow. First, ADAM12-associated stromal activation is most convincingly linked to physically restrictive architecture through ECM remodeling, collagen/fibronectin-rich desmoplasia, MMP14 and integrin-associated matrix regulation, vascular compression, elevated interstitial pressure, and impaired therapeutic access. Second, ADAM12-linked hypoxic and metabolic adaptation is supported by HIF- or Notch-linked ADAM12 induction and ADAM12-dependent HB-EGF/EGFR/FAK or HK1-associated signaling in defined tumor-cell models, whereas links to lactate accumulation, acidosis, and reverse Warburg metabolism operate largely through tissue-level or stromal-state programs. Third, ADAM12-associated immune-excluded or otherwise immunosuppressive states reflect multicompartment tissue remodeling that includes spatial immune positioning, myeloid organization, lymphatic and TDLN biology, and tissue-resident immune programs.

This framework carries a translational implication. ADAM12 may have value as a tissue or biofluid biomarker that helps identify barrier-dominant tumor ecosystems and, in selected settings, as a candidate entry point for stromal priming. ADAM12-informed intervention should be guided by isoform, cell-of-origin, spatial context, pathway activity, and direct or strong mechanistic evidence for an ADAM12-linked barrier program. This supports biomarker-guided stromal normalization and discourages broad stromal ablation, empirical ADAM12 inhibition, or ADAM12-only treatment selection.

Future studies should prioritize isoform-aware and cell-of-origin-aware profiling, spatial mapping, functional perturbation, and longitudinal monitoring. Mechanistic studies should separate direct ADAM12 perturbation evidence from mechanistic support, correlative human datasets, and inferred tissue-level links. This is especially important for lactate-driven immune remodeling, ILC2/eosinophil biology, type 2 immunity, lymphatic drainage, TDLN priming, and tissue-resident immune-cell states, which are important for modern TME biology but remain only partly ADAM12-specific.

The most productive way forward is to use ADAM12 as a context-resolved marker and organizing node for defining high-barrier tumor states and matching them to rational combinations. ADAM12 is not a universal single-molecule cause of every stromal, metabolic, lymphatic, or immune-resistance feature. Its mechanistic value depends on tumor context and evidence type. ADAM12-centered biology is most likely to become clinically useful when isoform-aware assays, spatial barrier profiling, cell-of-origin mapping, functional perturbation, and dynamic biofluid monitoring are integrated into the same translational workflow.

## Data Availability

No datasets were generated or analysed during the current study.

## Abbreviations

ADAM12: A disintegrin and metalloproteinase 12
CAF: Cancer-associated fibroblast
ccRCC: Clear cell renal cell carcinoma
CD147: Basigin
DC: Dendritic cell
ECM: Extracellular matrix
EGFR: Epidermal growth factor receptor
EMT: Epithelial–mesenchymal transition
ERK: Extracellular signal-regulated kinase
FAK: Focal adhesion kinase
HB-EGF: Heparin-binding EGF-like growth factor
HIF: Hypoxia-inducible factor
HK1: Hexokinase 1
IFN-I: Type I interferon
ILC2: Group 2 innate lymphoid cell
LUAD: Lung adenocarcinoma
MDSC: Myeloid-derived suppressor cell
MMP14: Matrix metallopeptidase 14
MSI: Microsatellite instability
MSC: Mesenchymal stromal cell
PDAC: Pancreatic ductal adenocarcinoma
SCLC: Small-cell lung cancer
TAM: Tumor-associated macrophage
TDLN: Tumor-draining lymph node
TGF-β: Transforming growth factor-β
TLS: Tertiary lymphoid structure
TMB: Tumor mutational burden
TNBC: Triple-negative breast cancer
YAP/TAZ: Yes-associated protein/transcriptional coactivator with PDZ-binding motif
